# Zn–IMP 3D Coordination Polymers for Drug Delivery: Crystal Structure and Computational Studies †

**DOI:** 10.3390/polym18010119

**Published:** 2025-12-31

**Authors:** Hafiz Zeshan Aqil, Yanhong Zhu, Masooma Hyder Khan, Yaqoot Khan, Beenish Sandhu, Muhammad Irfan, Hui Li

**Affiliations:** 1 Key Laboratory of Cluster of Science of Ministry of Education, School of Chemistry and Chemical Engineering, Beijing Institute of Technology, Beijing 100081, China; hafizzeeshanaqil@gmail.com (H.Z.A.); masoomakalyani@gmail.com (M.H.K.); yaqootkhan92@yahoo.com (Y.K.); 2 School of Pharmacy, North China University of Science and Technology, 21 Bohai Road, Caofeidian Xincheng, Tangshan 063210, China; zhuyh@ncst.edu.cn; 3 Department of Zoology, University of Sialkot, Sialkot 51040, Pakistan; benishsandhu17@gmail.com

**Keywords:** coordination polymers, Zn–IMP, hydroxyurea binding, targeted drug delivery, pH-dependent molecular dynamics

## Abstract

Coordination polymers (CPs) are garnering attention in the field of medicine day by day. The goal is to develop a CP with biosafe and environment-friendly characteristics. Herein, we report two such novel 3D coordination polymers of zinc-inosine-5′-monophosphate (Zn-IMP) and bpe/azpy (as linkers) which were engineered as metal–organic frameworks that can be used as drug carriers for hydroxyurea (HU). We employed SCXRD, PXRD, solid-state CD, FTIR and TGA for crystal structure characterizations; the results achieved 3D coordination polymers which contain a *P2*_1_ space group with chiral distorted tetrahedral geometry. Solution phase studies like UV–vis and CD were carried out to understand mechanistic pathways for interaction and chirality, respectively. We have also performed computational studies to evaluate the drug delivery capacity of both 3D CPs. Molecular docking and multi-pH molecular dynamics (MD) quantify that HU binds more strongly with CP−1 (ΔG =−10.87 ± 0.12) as compared to CP−2 (ΔG = −7.59 ± 0.26 kcal·mol^−1^), at normal and basic pH. MD simulation analysis indicated that a more compact and rigid cavity is observed by CP−1 as compared to CP−2 at physiological pH. Across acidic pH, for CP−1 the ligand RMSD increases markedly and U becomes slightly less negative, which indicated partial loss of contacts, thus releasing drugs in a tumor-like environment more easily. These result showed that CP−1 offers stronger binding, higher structural stability and a more pronounced pH-responsive release profile than CP−2, making CP-1 more promising candidate for targeted HU drug delivery, while CP−2 may serve as a weaker-binding, faster-release complement.

## 1. Introduction

Coordination polymers (CPs) that embed nucleotide ligands are emerging as versatile chiroptical carriers because they couple architecturally defined metal ligand scaffolds with stereochemical information encoded in biomolecular motifs [1]. Among available nucleotides, inosine 5′-monophosphate (IMP) is particularly well suited due to its ribose-nucleobase-phosphate triad which forms spatially separated donor sites that can interact with Zn(II), making dative bonds while simultaneously forming hydrogen-bonding and π-stacking surfaces for guest recognition [2,3]. When IMP-based nodes are elongated by linear N-donor spacers such as 1,2-bis(pyridin-4-yl)ethene (bpe) or 4,4′-azopyridine (azpy), the resulting frameworks can orient into axially helical assemblies whose Cotton effects sensitively report changes in environment and occupancy [3,4]. Chemotherapy mainly involves targeting cancerous tissues which are characterized by an acidic microenvironment typically exhibiting extracellular pH values ranging from 5.5 to 6.5, compared to the physiological pH of 7.4 in normal tissues [5,6,7,8,9]. This acidity arises primarily from the Warburg effect, where cancer cells preferentially undergo glycolysis even under aerobic conditions, leading to excessive lactic acid production [10,11]. Such a distinct pH difference provides an opportunity to design pH-responsive drug delivery systems that selectively release therapeutic agents within the tumor environment while minimizing off-target side effects [12,13,14]. Despite various nanocarrier platforms, CPs including metal–organic frameworks (MOFs) have emerged as highly promising candidates for targeted drug delivery due to their tunable porosity [15], high surface area and chemical versatility [16,17]. MOFs can be engineered to encapsulate anticancer drugs and respond to pH variations, remaining stable at physiological pH but flexible in opening their pores under acidic (tumor) conditions [18,19], thereby achieving site-specific drug release [17,20,21]. Over the past decade, extensive research has focused on developing biocompatible, stimuli-responsive MOFs such as ZIF-8, MIL and UiO-series frameworks for cancer therapy [22,23]. Many of these studies have demonstrated enhanced drug-loading capacities, controlled release kinetics and improved cytotoxic selectivity towards tumor cells [24,25,26]. Regardless of these properties, initial rapid burst release of drugs remains a significant drawback in the applicability of MOFs.

Hydroxyurea (HU), a compact, neutral to weakly acidic chemotherapeutic and free radical modulator, provides a useful test guest because its targeted release remains a significant challenge in biomedical interest [12,27,28,29]. A platform that couples HU binding with tunable and predictable multi-pH behavior would thus connect structural design to functional output in a directly measurable way [30,31]. Previously, our group has synthesized a nucleotide-based 3D MOF incorporating dTMP and azpy, and further evaluated the pH-dependent drug delivery profile of the synthesized MOF by using a doxorubicin as a drug candidate [32].

Despite notable advances in nucleotide-based CPs, fundamental questions remain unresolved [33]. First, how do IMP-anchored Zn^2+^ networks propagate and stabilize chirality in the presence of secondary linkers having a linear backbone structure with a different electronic character? Second, to what extent does secondary linker binding perturb circular dichroism (CD) and UV–vis line shapes and how are these spectral signatures linked to microscopic dynamics within the pores? Third, can such networks stably host HU across physiologically relevant pH windows (7.4 and 9.0) while providing targeted drug delivery at acidic pH (4 to 6) without sacrificing structural integrity? Answering these questions requires rigorous structure–property correlations involving the following: (i) single-crystal X-ray diffraction to establish absolute configuration and connectivity; (ii) bulk property characterizations by PXRD, FT-IR and TGA to verify phase purity and thermal resilience; and (iii) solution and solid-state CD and UV–vis spectroscopy to read out chiral and electronic environments. Equally, modern simulation approaches are needed to quantify pH-coupled stability, and to map contact fingerprints during HU binding that acts as a bridge from static crystal structures to dynamic behavior in fluid media and thus targeted drug delivery [2,34,35,36,37].

Guided by these gaps, we test the hypothesis that IMP-Zn nodes expanded by either bpe or azpy will yield coordination polymers (CP−1 and CP−2) with a helical bias and pore-surface chemistry that support a specific geometry [38,39]. We further postulate that the binding of secondary linkers will induce reproducible changes in CD and UV–vis line shapes, enabling spectroscopic tracking of incorporated linkers. Because azpy offers a larger π-surface and greater conformational rigidity than bpe, we anticipate distinct guest-recognition patterns and dynamic stability of CP with HU. We further anticipate that owing to the larger π-surfaces and greater conformational rigidity of azpy in CP−2, better guest recognition is presumed in molecular dynamic studies. Targeting cancer cells specifically is critical to reduce systemic toxicity, minimize side effects and enhance therapeutic efficiency. By exploiting the acidic tumor microenvironment, pH-responsive CPs enable precision drug delivery, ensuring that potent chemotherapeutic agents act primarily on malignant cells while sparing healthy tissues.

Our rationale rests on the complementary roles of the components. IMP contributes as a chelating/bridging phosphate–ribose–base manifold that anchors Zn^2+^ while transmitting chiral information into the extended network [40,41]. The bpe and azpy spacers enforce axial alignment and extend porosity, creating π-rich channels that favor HU recognition through N–H···O and O–H···O hydrogen bonds, dipolar contacts and dispersion. Zn(II), redox-inactive under the employed conditions, supports robust coordination at room temperature, permitting clean comparisons between crystallographic metrics and emergent properties such as CD response, thermal stability and pH-dependent binding. For the anticancerous drug delivery property of the CP−1 and CP−2, hydroxyurea was selected as a model drug, since it is administered to the cancerous patients as the first line of action. The mechanism of the HU is attributed to its antimetabolitic nature, which is administered orally, and it can cross the cell membrane easily due to its polar nature [42]. Apart from that, the ADMET properties show it to be a good drug candidate as it also follows Lipinski’s rule of five [43,44,45]. The drawback lies in the fact that when HU is given for a long time, it itself can cause leukemia since it affects all cells equally. Guided by this drawback, the current study aims to do the targeted drug delivery. Finally, docking and MD simulations with explicit protonation state targeted drug delivery provide pose-resolved, time-averaged pictures of HU capture that rationalize chiroptical readouts and deconvolute the respective roles of linker electronics and rigidity.

## 2. Materials and Methods

All reagents were used as received unless otherwise noted. The spacers 1,2-bis(pyridin-4-yl)ethene (bpe) and 4,4′-azopyridine (azpy) were purchased from Meryer, Zn(II) nitrate (Zn(NO_3_)_2_) and zinc trifluorooxalate Zn(C_2_O_4_F_3_)_2_ were obtained from Alfa Aesar and inosine 5′-monophosphate disodium salt (IMP·2Na) was obtained from Alfa Aesar. Hydroxyurea (HU) was of analytical grade. Ultrapure water (18.2 MΩ·cm at 25 °C) was used for all preparations, and nitric acid, HNO_3_ (1 M) served for pH adjustment. FT-IR spectra were recorded on a Nicolet Nexus instrument (Madison, WI, USA) using KBr pellets over 4000–400 cm^−1^. UV–vis spectra were acquired on a TU-1950 spectrophotometer (PuXi, Shaanxi, China) using 1 cm quartz cuvettes with baselines recorded against solvent blanks. Powder X-ray diffraction (PXRD) was performed on a Bruker D8 Advance diffractometer (Bruker AXS GmbH, Karlsruhe, Germany) using Cu Kα radiation (λ = 1.5406 Å) at room temperature, typically scanning 5–50° in 2θ with a step size of 0.02°. Single-crystal X-ray diffraction (SCXRD) employed Bruker APEX-II CCD and Rigaku Saturn724+ (2 × 2 bin mode) detectors with graphite-monochromated Mo Kα radiation (λ = 0.71073 Å). Circular dichroism (CD) spectra were collected on a JASCO J-810 spectropolarimeter (Jasco, Tokyo, Japan) under continuous nitrogen, scanning at 50–100 nm·min^−1^ and averaging at least three accumulations in 1 cm cells unless stated otherwise. Thermogravimetric analysis (TGA) was carried out on a Shimadzu DTG-60H (Shimadzu, Kyoto, Japan) from 30 to 800 °C at 5 °C·min^−1^ under nitrogen (~50 mL·min^−1^). Sample pH was measured with a PHS-3C meter (Yoke, Shanghai, China) after three-point calibration at pH 4.00, 7.00 and 10.00 at 25 °C. Standard PPE (gloves, lab coat and eye protection) was used throughout, and nitric acid and zinc salts were handled in a fume hood; waste containing Zn^2+^ and nitrate was collected according to institutional protocols. To ensure reproducibility, all synthetic reactions were repeated at least three times with yields consistent within ±3% absolute. Instrumental calibrations were verified daily, including PXRD 2θ zero checks with a silicon standard, CD baselines and HT voltage limits, and UV–vis wavelength accuracy using a holmium oxide filter.

### 2.1. Synthesis and Structural Characterization

**Scheme 1:**
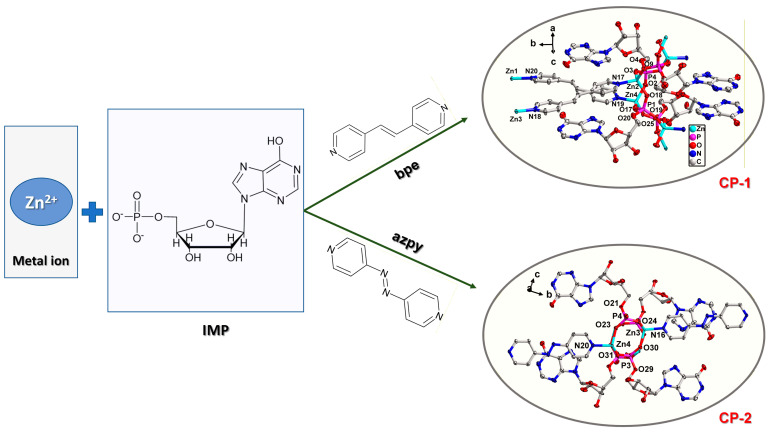
MOF preparation using IMP (nucleotide) and auxiliary ligands (bpe/azpy), creating two coordination polymers, CP−1 and CP−2.

For assembly of CP−1 and CP−2, separate aqueous solutions of IMP (as the disodium salt) and Zn(C_2_O_4_F_3_)_2_ were prepared by dissolving each reagent in 5 mL deionized water at room temperature (22–25 °C) under gentle stirring (~400 rpm). The two solutions were then combined and stirred for approximately 20 min, giving a colorless clear mixture consistent with rapid formation of IMP-Zn oligomeric nodes. A 5 mL aqueous solution of the chosen spacer—bpe for CP−1 or azpy for CP−2—was subsequently added dropwise over 2–3 min and stirring was continued for an additional 30 min. If slight opalescence appeared at this stage, the suspension was carefully acidified with 1 M HNO_3_, a step that reproducibly promoted dissolution of colloidal intermediates and favored growth of high-quality crystals. After a further 30–40 min of stirring, the reaction mixture was passed by gravity through a 0.45 µm PTFE membrane to remove dust or unintended seeds. The clear filtrate was then allowed to evaporate slowly at room temperature in loosely covered vials. Over several days, colorless single crystals suitable for SCXRD were obtained. The isolated yield calculated on zinc was typically about 35%. Bulk polycrystalline fractions were also harvested by gentle decantation of mother liquor followed by air-drying (Scheme 1).

Bulk and single-crystal consistency was verified by PXRD: simulated patterns generated from the refined SCXRD models matched experimental PXRD profiles for both CP−1 and CP−2, confirming phase purity and structural fidelity at scale (Figure S17). Thermal and spectroscopic checks further supported the assignments. TGA established the lattice and coordination water content and the thermal decomposition sequence (Figure S21), while FT-IR spectra tracked characteristic ν(P=O), ν(P–O), ν(C=N) and ν(C=C) bands and Zn–O/N lattice modes, revealing shifts diagnostic of nucleotide coordination and spacer binding (Figure S18).

Chiral responses were evaluated by CD spectroscopy using aqueous dispersions or fine suspensions of CP−1 and CP−2 adjusted to a typical optical density of 0.3–0.8 at the strongest absorption band. Observed Cotton effects were interpreted in the context of the structural parameters derived from crystallography, thereby relating axial bias. When comparing CD and UV–vis data across conditions, ionic strength was held constant to avoid confounding electrostatic effects.

### 2.2. Single-Crystal X-Ray Diffraction: Data Collection and Structure Solution

Single crystals of suitable size and quality were selected and mounted under ambient conditions. Data for representative crystals were collected at 296.15 K on a Bruker DUO APEX II-CCD using graphite-monochromated Mo Kα radiation (λ = 0.71073 Å), with generator settings of 45 kV and 30 mA. Unit-cell parameters were obtained and reflections were indexed using the XSCAN routine, and intensities were collected with an ω–2θ scan strategy to ensure adequate redundancy and coverage. Absorption effects were treated by empirical multi-scan correction. Initial structure solutions were obtained using SHELXT within the Olex2 interface, and full-matrix least-squares refinement on F^2^ was performed with SHELXL. Non-hydrogen atoms were located from Difference Fourier Maps and refined anisotropically. Hydrogen atoms attached to carbon and nitrogen were placed in calculated positions and refined using riding models, whereas hydrogen atoms associated with lattice water were included when visible in residual maps or were restrained to chemically reasonable geometries. Final residuals and goodness-of-fit statistics were within accepted limits for the refined space groups. Key crystallographic parameters and CCDC deposition numbers are summarized in Table 1. Additional experimental and structural details for CP−1 and CP−2 are reported in significant bond lengths, angles and hydrogen bonding (Tables S1–S6). The refined structures were used to simulate PXRD patterns for direct comparison to experimental data, thereby linking the single-crystal models to bulk characterization. To further verify bonding interactions, Hirshfield analysis was performed using CrystalExplorer 17.5 [46].

### 2.3. Computational Modeling: Docking, Interaction Fingerprints, and Multi-pH Molecular Dynamics

To rationalize HU binding under explicit pH control and to complement the experimental characterizations, we employed an MOE-based (2009 release) docking-plus-MD workflow. Oligomeric segments of CP−1 (IMP-Zn-bpe) and CP−2 (IMP-Zn-azpy) capturing the coordination environments and accessible pore surfaces observed by SCXRD were extracted and end-capped with neutral groups that preserved local geometry. All structures were prepared in Molecular Operating Environment (MOE) 2009, and protonation states for both frameworks and HU were assigned using Protonate3D at target pH values (5.0, 7.4 and 9.0) with manual inspection of phosphate and nucleobase sites to ensure chemically sensible states in the presence of Zn^2+^. Amber 10:EHT partial charges were applied and Zn^2+^ coordination spheres were retained as in the experimental models.

Docking preceded MD to generate plausible starting poses. HU was placed within the framework segments using Triangle Matcher with London dG prescoring, followed by force-field/implicit-solvent refinement and finally by GBVI/WSA dG rescoring. We report the MOE Score S (E_score2) and pose-level ΔG estimates. Per-pose contacts including hydrogen bonds, π-π interactions, cation-π contacts where relevant and other polar interactions were exported from MOE Ligand Interactions panel to generate interaction fingerprints. These docking results informed the selection of representative starting geometries for MD and established preliminary hypotheses for contact motifs expected to persist in explicit dynamics.

For MD, each polymer-HU complex was simulated for 100 ns, saving 10,000 frames (10 ps per frame). For CP−1, trajectories were computed at pH 5.0, 7.4 and 9.0; for CP−2, a representative physiological condition was examined. The MD environment used MOE force-field implementation with an implicit solvent model compatible with GBVI/WSA scoring. To preserve the experimentally observed Zn-O/N coordination while allowing natural breathing modes, framework-heavy atoms in the immediate coordination sphere were minimally restrained during equilibration and then released to weak restraints during production. For each trajectory, MOE reported RMSD for receptor and ligand, Rg, solvent-accessible surface area (SASA) and energies (total potential U, an enthalpy proxy H and kinetic K) per frame. To quantify stability, the equilibrated window was defined a priori as the final 20% of frames (frames 8001–10,000) and mean ± SD values were computed for RMSD, Rg, SASA, U, H and K to enable fair comparison across pH and polymer identity.

To connect time-averaged metrics with concrete geometries, five representative snapshots per system frames 1, 2500, 5000, 7500 and 10,000 were analyzed. Interatomic distances between HU donors/acceptors and framework sites (phosphate oxygen, nucleobase nitrogen/oxygen, spacer nitrogen and Zn-coordinated donors) were measured and collated into a pose-contact digest. This digest tabulates counts and fractions of strong contacts (≤2.70 Å) and moderate contacts (2.71–3.20 Å), complementing the interaction fingerprints and identifying persistent hydrogen-bonding and π-stacking registers that likely underpin the experimentally observed chiroptical modulations. By aligning docking-suggested motifs, pose-digest statistics and plateau values from RMSD, Rg and SASA within the equilibrated window, we established whether HU engages the same binding patches that are structurally plausible from SCXRD and spectroscopically implicated by CD/UV–vis.

### 2.4. Data Handling and Analysis

Trajectory equilibration was assessed by inspection of RMSD traces to confirm stabilization, operationally defined as a drift below 0.1 Å over at least 10 ns within the production segment. In cases where larger fluctuations were observed, most often at the highest pH for CP−1, we verified that these variations corresponded to reversible breathing motions rather than irreversible coordination changes; under the applied restraints, no Zn-O/N bond rupture events were detected. Reported averages are presented with standard deviations computed over the equilibrated window (n = 2000 frames per 100 ns run). When comparing conditions, differences in means were discussed together with effect sizes (Cohen’s d) to convey practical significance even in the absence of formal hypothesis testing. Spectral data were processed conservatively; CD spectra were only smoothed when explicitly noted (Savitzky–Golay, second order, seven-point window) without altering band positions. UV–vis spectra were baseline-corrected without deconvolution unless specified. Where appropriate, HU binding isotherms quantified from CD band areas or UV–vis difference spectra as a function of HU equivalents were fit to 1:1 or 1:n site models to estimate apparent K_d_ values with fits and residuals reported.

PXRD profiles measured experimentally were compared to patterns simulated from the SCXRD structures using identical wavelengths and consistent peak shape parameters. Minor preferred-orientation effects associated with needle-like habits were evaluated qualitatively by monitoring the relative intensities of hkℓ reflections known to be orientation-sensitive. TGA mass-loss steps were assigned to lattice/coordination water and organic decomposition by correlating step temperatures with diagnostic FT-IR changes such as the loss of ν(OH) features and the emergence or attenuation of ν(C=N) and ν(P-O) bands in ex situ spectra of partially heated aliquots when these were examined. Derived (equilibrated-window statistics and pose-contact digests) raw MD time-series plots (RMSD, Rg, SASA and energies).

### 2.5. Controls, Calibration and Additional Considerations

Blank and matrix controls were implemented to isolate framework-specific effects. IMP with Zn^2+^ in the absence of spacer was examined by FT-IR and PXRD to confirm that the emergence of axial chirality and amplified signals requires extension by bpe or azpy. HU in buffer produced no CD within the relevant spectral window, ensuring that observed Cotton effects originate from CP–HU interactions. Reversibility under pH cycling (5.0 → 7.4 → 5.0) was assessed by CD to test for non-destructive binding. For spectroscopy, sample concentrations were adjusted to maintain absorbance between approximately 0.4 and 0.6 at the principal UV band in 1 cm cells to balance signal quality and linearity. All spectroscopic and TGA measurements were collected in triplicate with representative traces presented in the main text and replicate envelopes provided in the SI where clarity allowed. Instrumental uncertainties were propagated where applicable (pH ±0.02 units at 25 °C temperature ramp accuracy ±0.2 °C) and these considerations were integrated into the interpretation of condition-dependent trends.

## 3. Results and Discussion

### 3.1. Design and Synthesis

The design foundation was to embed the inherently chiral inosine 5′-monophosphate (IMP) nodes within Zn(II) coordination environments and to propagate axial bias by threading linear N-donor linkers of comparable length 1,2-bis(pyridin-4-yl)ethene (bpe) or 4,4′-azopyridine (azpy) across phosphate-bridged zinc columns. This strategy aimed to couple the ribose-encoded stereochemical information with π-rich channels, the curvature and hydrogen bond topology of which could be tuned by the auxiliary linker while keeping the nucleotide geometry largely unchanged. Slow evaporation from mildly acidified aqueous media, reproducibly, afforded colorless single crystals and phase-pure polycrystalline solids for both materials. As anticipated from the matched linker lengths and common IMP-Zn nodes, powder and single-crystal X-ray diffraction established that CP−1 (IMP-Zn-bpe) and CP−2 (IMP-Zn-azpy) are isostructural at the level of their inorganic organic scaffolds, differing primarily in the nature of the linear co-linker that spans Zn(II).

### 3.2. Crystal Structure

**Figure 1:**
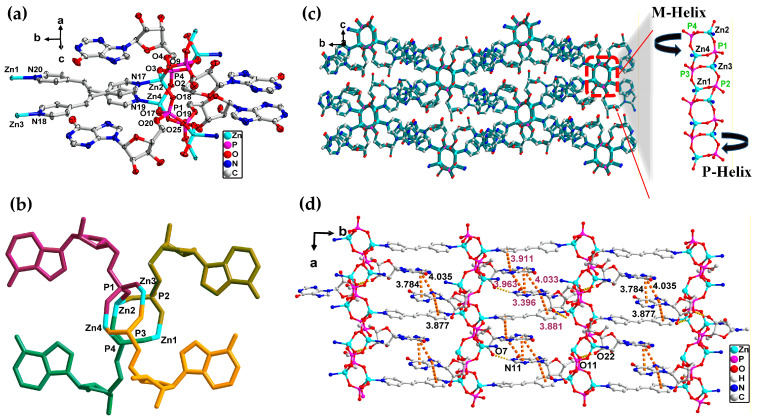
(**a**) Coordination environment of the Zn1-Zn4 center atoms of CP−1. (**b**) Coordination environment of the Zn1-Zn4 cluster of CP−1 connects with the phosphate atom of the IMP (H and auxiliary ligands are removed due to clarity). Hydrogen atoms and uncoordinated water molecules are removed for clarity. (**c**) Schematic presentation of the 3D architecture of CP−1 down-viewed from a-axis and perspective view of the axial coordination and bridging mode of oxygen atom of the phosphate group and the central Zn(II) ion. (**d**) 3D coordination polymer of CP−1 formed by two different bpe ligands connecting the zinc–phosphate chains down-viewed from c-axis, as well as the abundant hydrogen bonds and multiple π-π stacking interactions in this 3D structure.

CP−1 and CP−2 are isostructural and we take CP−1 as an example for detailed analysis (Figure S17 and Table 1). Detailed analysis of CP−1 is illustrative of the structural motif common to both frameworks. CP−1 crystallizes as a three-dimensional chiral coordination polymer centered on Zn(II) ions in the monoclinic system with chiral space group *P2*_1_. The asymmetric unit contains four IMP ligands, two bpe ligands, four Zn centers and fourteen guest water molecules. Each zinc ion adopts a distorted tetrahedral N/O coordination sphere composed of one pyridyl nitrogen from a bpe and three non-equivalent phosphate oxygens from three distinct IMP ligands, resulting in four-coordinate Zn-N, Zn-O environments (Figure 1a,b). Extension via the three IMP phosphate donors along the a-axis generates one-dimensional columns in which alternating Zn and O(P) atoms close into eight membered Zn-O-P-O rings that oscillate between boat and chair conformations (Figure S2). Superimposed on this axial growth, the auxiliary linkers (bpe) bridge laterally and connects zinc nodes across the b/c planes to create a double bridge two-layer motif (Figure 1c). Two crystallographically distinct bpe molecules coordinate Zn1/Zn2 in one layer (layer 1) and Zn3/Zn4 in the opposing layer (layer 2) such that the layers are related by their opposite propagation along the c-axis and their interdigitation yields a 3D chiral network (Figure 1d).

Within this architecture, the nucleotide components are conformationally supported by a dense lattice of self-complementary hydrogen bonds among pentose hydroxyls, phosphate oxygens and purine heteroatoms (O22-H22C⋯O11 and N11-H11⋯O7 (Table S3). These interactions help stabilize the flexible sugar phosphate manifold while the π-surfaces of purines align favorably with nearby bpe rings. The axial propagation generates an extended axial chirality (EAC) in which four nucleotides organize as two counter rotating groups along the Zn-bpe-Zn-P-Zn-bpe-Zn axis; one group rotates clockwise and the other counterclockwise (Figure 1c). This alternation produces four distinct zinc sites and a repeating Zn⋯Zn metric of 13.32 and 13.33 Å. To satisfy the orientations of the four IMP ligands, the two adjacent bpe linkers adopt different torsional states reflected in dihedral angles of 21.01° and 5.75°, respectively, underscoring that small linker conformational adjustments accommodate the chiral packing demand imposed by IMP (Figure S1).

Face to face arrangement of purines is a pervasive stabilizing element. Along the c-axis, purine bases display close to parallel relationships both with each other and with bpe, giving rise to multiple π-π stacking contacts with centroid separations clustered near 3.39, 3.78, 3.96 and 4.03 Å (3.88, 3.39, 3.91, 3.96, 4.03 Å and 3.78, 3.88, 4.03 Å). Between Zn1 and Zn2, both nucleotides engage in quadruple π-π stacking per purine ring and additionally interact with neighboring bpe ligands, whereas between Zn3 and Zn4, one IMP exhibits threefold stacking and the other twofold, indicating layer-dependent π overlap (Figure 1d). The pentose conformations track these packing differences: the two IMPs between Zn1 and Zn2 adopt envelope ^2^E and ^3^E forms between Zn3 and Zn4, the doubly stacked IMP favors ^3^E, while the triply stacked IMP adopts a ^2^T_3_ conformation (Table S7) [38]. Two factors plausibly bias this T-form. First, the doubly stacked purine shows negligible π-π contact to nearby bpe, making its conformation rely primarily on purine-purine stacking, which confers greater sugar flexibility. Second, for the triply stacked IMP, the pentose hydroxyl does not participate in external hydrogen bonds, unlike the other three nucleotides, whose hydroxyls do engage nearby phosphate or base heteroatoms (O31–H31E⋯O24) (Table S3). Loss of this H-bonding constraint plausibly enables the ^2^T_3_ puckering. The structural geometry resembles an SCU-type MOF.

Collectively, phosphate directed Zn-O coordination along the a-axis double bpe bridges across the b- and c-axis and a cooperative H-bond/π-stacking network formed a rigid axially biased 3D motif (Figure 1d). The same topology is realized in CP−2 when bpe is replaced by azpy, as shown in Figure 2. CP−1 and CP−2 are iso-structural according to the PXRD/SCXRD (Figures S8–S17) and Table 1.

### 3.3. UV–Vis Spectroscopy

**Figure 3:**
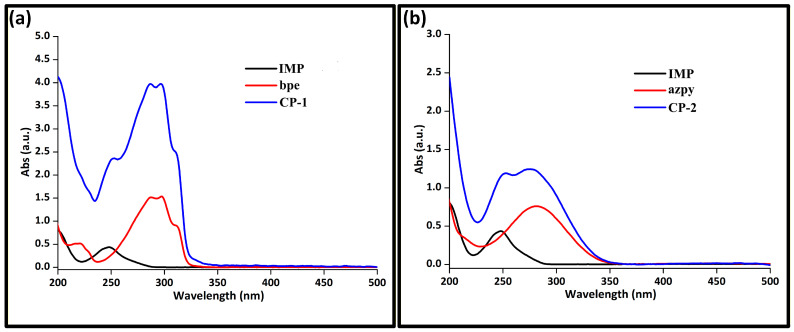
(**a**) The solution-state UV–vis absorption spectra of IMP, bpe and CP−1. (**b**) The solution-state UV–vis absorption spectra of IMP, azpy and CP−2. The spectra were obtained by measuring 2.5 × 10^–5^ mol·L^–1^ solution in a 1 cm cell.

Electronic absorption spectra corroborate the structural narrative by reporting on the relative contributions of nucleotide and linker chromophores within the frameworks. For IMP, the trough near 222 nm is assigned to an n-π* transition centered on nucleobase heteroatoms, and the prominent band at 247 nm corresponds to π-π* excitation of the purine ring [47,48,49,50]. Both features remain evident in CP−1 and CP−2 but they are systematically shifted by framework assembly. In CP−1, the bpe ligand contributes a downward slope between 209 and 236 nm (n-π*) and a broad envelope extending into 250–323 nm (π-π*) such that the composite spectrum shows shoulders at 222 and 230 nm (n-π*) and at 250, 285, 298 and 311 nm (π-π*) [51]. Notably the 230, 285, 298 and 311 nm features coincide closely with those of free bpe, indicating that these maxima largely arise from bpe-localized transitions that survive coordination while being modulated by the IMP-Zn environment (Figure 3a). CP−2 follows the same logic with azpy; a clear new maximum at 276 nm appears attributable to the azpy π-π* transition while a trough near 232 nm corresponds to its n-π* band (Figure 3b) [52]. Relative to the isolated ligands, both CPs display red shifted nucleotide n-π* and π-π* transitions consistent with increased conjugation and environmental polarization within the framework, while conversely the linker-dominated bands are slightly blue shifted compared to the free linkers, consistent with coordination-induced perturbations that reduce the effective conjugation length of the linker moiety within the solid. The co-existence of ligand-inherited and framework-emergent bands is therefore diagnostic of successful incorporation of both chromophore classes and their non-trivial coupling in the assembled CPs.

### 3.4. Solution-State Circular Dichroism

**Figure 4:**
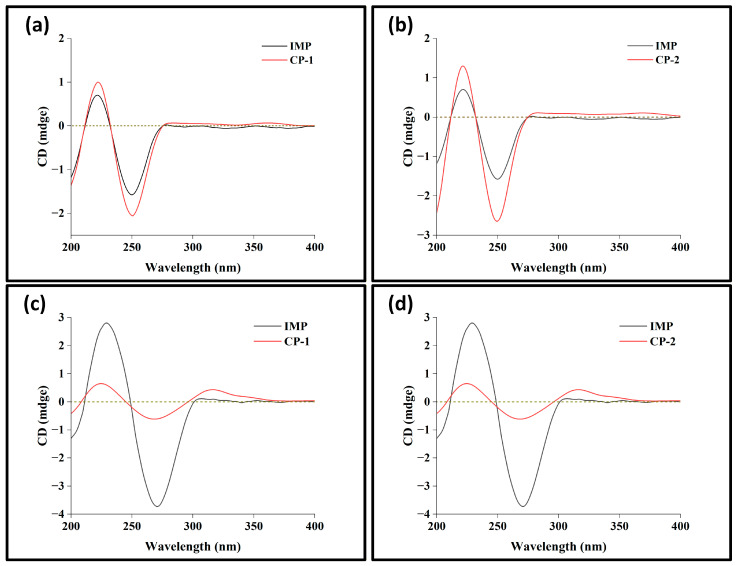
(**a**,**b**) The liquid-state CD spectrum of IMP, CP−1 and CP−2 in aqueous solution at room temperature; the spectra were obtained by measuring 2.5 × 10^–5^ mol/L solution in a 1 cm cell. (**c**,**d**) The solid-state CD spectrum of IMP, CP−1 and CP−2 in solid at room temperature; the spectra were obtained by measuring 2.5 × 10^–5^ mol/L solution in a 1 cm cell.

In aqueous solution, the CD signatures are dominated by the inherent chirality of the nucleotide rather than by the extended axial bias evident in the solid as expected for frameworks that partially dissociate into oligomeric species or expose monomeric chromophores under dilute conditions. The IMP ligand displays a positive Cotton effect near 222 nm (n-π*) and a negative one near 250 nm (π-π*) [38,53]. CP−1 and CP−2, when dispersed at comparable concentrations, manifest the same pair of features but with significantly enhanced magnitudes relative to free IMP (Figure 4a,b). We attribute this amplification primarily to coordination with Zn metal; the chromophore groups around the chiral center in the IMP is enhanced because of the structural rigidity; correspondingly, coordination to Zn(II) biases the population toward the β-type and restricts the conformational freedom of IMP to undergo puckering, thereby increasing the rotatory strength of the n-π* and π-π* bands [54,55]. The close similarity of the solution CD spectra for CP−1 and CP−2 is consistent with their isostructural cores and with the linker chromophores contributing minimally to solution-phase chirality under conditions where extended axial correlations are not preserved.

### 3.5. Solid-State Circular Dichroism

In the solid state, where the extended lattice is preserved, chiropal readouts capture both sources of chirality ribose configuration and extended axial chirality (EAC) [56,57]. Solid IMP exhibits two principal features, a band at 230 nm (n-π*) and one at 272 nm (π-π*) [38]. For CP−1 and CP−2, the sign pattern in the EAC-sensitive region is consistent with M-type helicity with nucleotides arranged counterclockwise around the M-bpe/azpy-M, which rationalizes the observed negative Cotton effect in the corresponding region (Figure 4c,d) [58]. A modest red shift of the positive band to 235 nm in both CPs is readily explained by stabilization of the n-π* transition through intra and intermolecular hydrogen bond networks in the lattice which lower the excited state energy and shift absorption to a longer wavelength. Interestingly, despite clear sugar-pucker differences in the crystallographic analysis (^2^E/^3^E vs. ^2^T_3_), the solid-state CD spectra do not present resolvable, sugar-specific signatures [38]. We assign this to spectral congestion and “chirality averaging”; multiple chiral sources (ribose at four sites per asymmetric unit helical EAC and π-π stacked purines) overlap in the 200–300 nm window such that small sugar-pucker perturbations are masked by the stronger EAC-driven exciton coupling and by linker-assisted anisotropy [2,59]. The Flack parameters near zero for both CPs confirm single-handed crystals and thus validate the assignment that the observed CD sign pattern reflects true macroscopic chirality rather than twinning or racemic intergrowth.

### 3.6. Chirality

Taken together, the diffraction and spectroscopy establish a coherent picture. Along the a-axis, phosphate-bridged Zn(II) columns generate eight membered rings whose conformational alternation transmits axial bias; across the b-axis and c-axis, bpe or azpy double bridges stitch columns into chiral layers that interpenetrate to produce a rigid 3D chassis. This architecture enforces parallelism between purines and linkers and seeds extensive π-π contacts while an H-bond mesh fixes pentose and phosphate orientations. The structural consequences are captured optically: solution CD magnifies intrinsic nucleotide chirality through coordination-restricted conformational space whereas solid-state CD reports the M-type EAC of the extended lattice with small red shifts attributable to H-bond stabilization. UV–vis fingerprints finally disentangle nucleotide and linker-centric transitions and demonstrate that both classes of chromophores survive and couple within the frameworks. That CP−1 and CP−2 remain isostructural yet show subtle spectral differences at 230–311 nm underscores the role of linker planarity and π-surface (ethylene vs. azo) in tuning band positions and intensities without disturbing the IMP-driven chiral scaffold.

### 3.7. FTIR Analysis

In CP−1, the vibrational modes of the purine base (pyrimidine and imidazole) ring framework are located at 1608 cm^−1^ and 1578 cm^−1^ (Figure S18a). Compared with the IMP ligand (1589 cm^−1^), these vibrational bands show significant splitting, indicating that in CP−1, the purine base may be involved in the formation of a new hydrogen-bonding network. The symmetric and asymmetric vibrations of the phosphate group in CP−1 are observed at 1099 cm^−1^ and 1016 cm^−1^, 993 cm^−1^, respectively. Compared with the ligand, the asymmetric vibrational absorption of the PO_3_^2-^ group (978 cm^−1^) undergoes splitting, while the symmetric vibrational absorption (1089 cm^−1^) shifts to higher frequencies, which may be due to the coordination of phosphate oxygen atoms with metal ions leading to a rearrangement of the hydrogen-bonding network. The infrared absorption spectra of complexes CP−1 and CP−2 show similar trends to that of the IMP ligand (Figure S18b).

### 3.8. TGA

The water content and thermal stability of CP−1 and CP−2 were estimated separately using thermogravimetric analysis (TGA). The test results are shown in Figure S19a,b. The TGA curves indicate that CP−1 and CP−2 undergo a two-step weight loss process. CP−1 and CP−2 lose 10.87% (calculated 11.18%) and 9.89% (calculated 11.16%) of their mass at 30–125 °C and 30–150 °C, respectively, corresponding to 14 free water and coordinated water molecules in their unit cells. As the temperature rises to 203 °C, 221 °C, 233 °C and 240 °C, the frameworks of CP−1 and CP−2 begin to collapse. The analysis of water content in CP−1 and CP−2 is almost consistent with the C, H and N, which further confirms the chemical structure determined by single-crystal X-ray diffraction.

## 4. In Silico Studies

### 4.1. Hirshfielf Surface Analysis

The calculations of intermolecular interactions for the developed coordination polymers were performed by using the CrystalExplorer 17.5 software [60] incorporating Hirshfield analysis and the 2D fingerprint plots [61]. The ratios of de vs. di were calculated by utilizing the denorm function curve (where de and di represent external and internal distances to the nearest nuclei). The red surfaces are the illustrations of short contacts or negative dnorm values which correspond to the N---H, H---H, O---H, and C---H contacts. The confirmation of the presence of the non-covalent interactions was performed by the Hirshfield surface plots (Figure S23).

Fingerprint plots generated for CP−1 and CP−2 show hydrogen bonds (Figures S24 and S25). The overall two-dimensional plots for CP−1 were delineated into C---H/H---C (6%), H---H (34.4%), N---H/H---N (10.8%), O---H/H---O (32.4%), Zn---N/N---Zn (2%) and Zn---O/O---Zn (2.7%). Similar, hydrogen bond interactions were observed for CP−2 [61]. Computational docking: pose energetics and contact topology.

**Figure 5:**
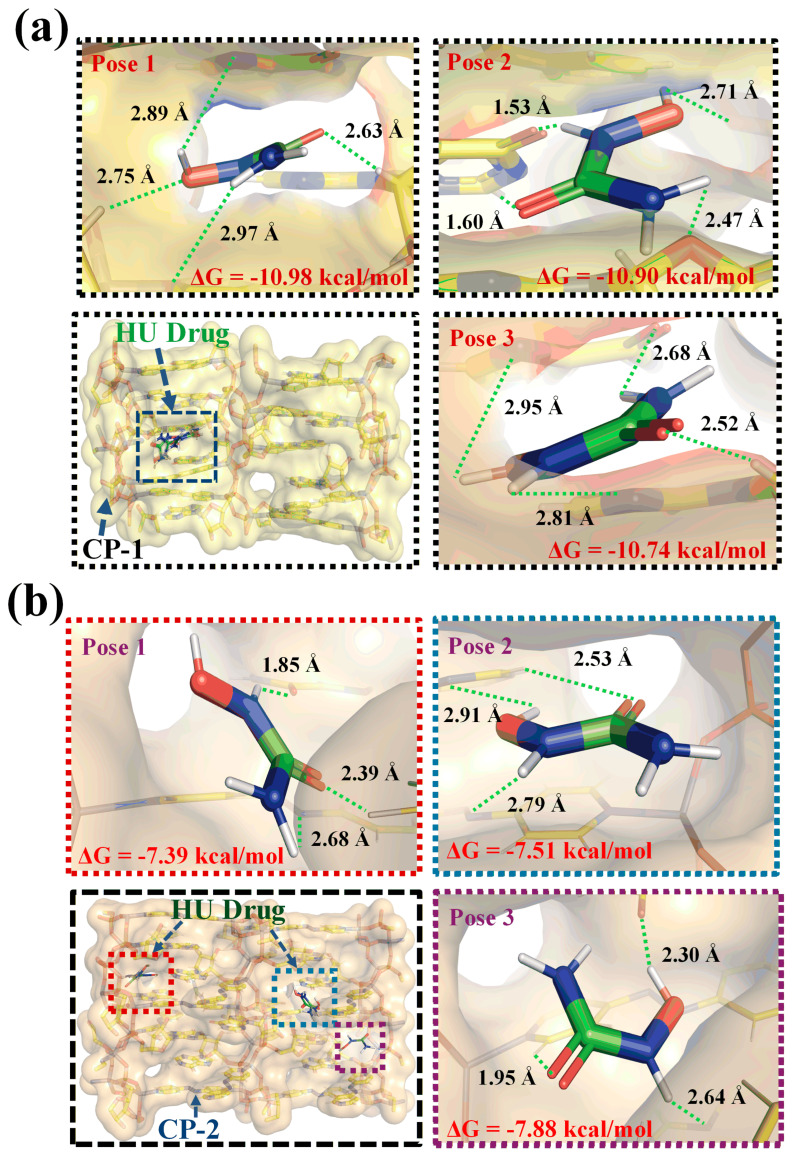
(**a**) Molecular docking of CP−1 with the drug of three different poses and it shows the hydrogen-bonding interaction of CP−1 with the drug in green dotted lines. (**b**) Molecular docking shows the three different poses of drug with CP−2 and the drug binding is in three different pockets. Green dotted lines show the hydrogen-bonding interaction and distance between drug and CP−2.

To connect static structure readouts to host–guest recognition, we docked HU into oligomeric segments extracted from the SCXRD models using MOE 2009. Despite differing score scales, both independent readouts, the annotated ΔG on three representative poses and the refined MOE score S over the top five poses, converge on the same rank order: CP−1 (IMP–Zn–bpe) binds more strongly than CP−2 (IMP-Zn-azpy). For CP−1, the representative poses return ΔG = −10.98, −10.90, −10.74 kcalmol^−1^ (mean ΔG = −10.87 ± 0.12) whereas CP−2 gives −7.39, −7.51, −7.88 kcal mol^−1^ (mean ΔG = −7.59 ± 0.26), a difference in ΔG ≈ −3.28 kcal·mol^−1^ favoring CP−1 and corresponding at 298 K to an ≈2.5 × 10^2^-fold affinity advantage. Consistently, across the top five refined poses, S_mean_ is more favorable for CP−1 (−3.771 ± 0.047; S_min_ = −3.832) than for CP−2 (−3.555 ± 0.125; S_min_ = −3.694) with similarly small RMSD_refine values (≈1.0–1.6 Å) attesting to well-behaved pose families (Figure 5a,b, Tables S8 and S9).

Quantitative contact fingerprints explain this energetic split mechanistically. In CP−1, HU frequently achieves multipoint engagement along the bpe-defined tunnel with two to four short contacts in the same orientation and sub-2.0–2.7 Å distances common (1.53–1.66, 2.47–2.71, 2.81–2.97 Å) in the three illustrated poses. Over all 28 poses analyzed, CP−1 exhibits H-bond counts per pose of 0 (57.1%), 1 (17.9%), 2 (7.1%), 3 (14.3%) and 4 (3.6%), and H-bond lengths (flagged Yes) center at a median 2.559 Å (Q1–Q3: 2.483–2.888, min-max 2.061–2.947). CP−2 presents a slightly wider more planar; although it can form very short individual contacts (down to ~1.85–1.95 Å in representative poses), concurrent multipoint anchoring is less frequent. Across 31 poses, the H-bond distribution is 0 (41.9%), 1 (35.5%), 2 (12.9%), 3 (6.5%), 4 (3.2%) with a longer median H-bond length of 2.710 Å (Q1-Q3: 2.575–2.833, min-max 2.077–2.987). Within 3.5 Å, CP−1 records 79 framework contacts enriched in N/O sites near the bpe channel C(26) N(18) H(18) O(17) whereas CP−2 shows 97 contacts distributed more evenly O(26) H(25) C(24) N(22). Thus, the decisive difference is not raw contact count but cooperativity; CP−1 more often assembles several moderate short donor acceptor contacts simultaneously in a single pocket while CP−2 more often distributes one to two contacts in wall-aligned shallower orientations. Empirical GBVI/WSA scoring rewards exactly this multi-bond convergence and explains the ~3.3 kcal·mol^−1^ advantage for CP−1.

### 4.2. Multi-pH Molecular Dynamics: Stability, Compactness and Retention

Time-dependent behavior underpins practical load/release. We therefore propagated 100 ns MOE 2009 trajectories (10,000 frames) for CP−1·HU at pH 5.0, 7.4 and 9.0 and for CP−2·HU at pH 5.0, 7.4 and 9.0, evaluating the equilibrated window (last 20%) for RMSD, radius of gyration (Rg), solvent-accessible surface area (SASA) and energies (U, H, K) (Figures S21 and S22). As summarized in Table 2, multi-pH simulation confirms structural stability over a 100 ns time frame. Both the polymers contradict each other slightly in their binding behavior at varying protonation states corresponding to the pH conditions. The RMSD of CP−1 is constrained over a small window of 3.0–3.5 A for all three pH variations, with the lowest value at pH 7.4 (3.02 ± 0.11 Å) indicating a rigid framework which shows equilibrated state. Similarly, CP−2 also shows stability (RMSD 3.38–3.74 Å) but with more fluctuations over the course of time. The Rg values complement the RMSD fluctuations and show a larger radius for CP−2 (~13.9 Å) as compared to CP−1 (~11.7 to ~11.9 Å), which reveals an expanded and conformationally flexible host to be CP−2 as compared to CP−1. These findings point CP−1 as being a more compact, shape-confinable cavity for HU encapsulation.

Not only this but the drug’s binding behavior with the polymer is pH-dependent. Extremely low values of RMSD exhibited by HU at pH 7.4 with both CP−1 (0.028 Å ± 0.01) and CP−2 (0.038 Å ± 0.01) show tight confinement of the drug in a well-defined binding pocket and minimal conformational and positional drift. At these levels, it is clearly seen that HU’s binding behavior with CP−1 is more pronounced as compared to CP−2. Upon acidification (mimicking tumor environment), the RMSD drug increases sharply for CP−1 (0.34 ± 0.05 Å) as compared to CP−2 (0.20 ± 0.02 Å). This obvious gain in mobility of HU within CP−1 illustrates disruption of host–guest contacts at acidic pH, allowing the drug’s release from the rigid framework.

The energy parameters also validate the above binding behavior. The interaction energy is most favorable for CP−1 at physiological pH, and least favorable for the acidic pH conditions. CP−2 shows a similar trend but the absolute interaction energies of CP−2 are less negative at all pH conditions as compared to CP−1, which consistently shows overall weaker binding. Thus, altogether, MD results suggest CP−1 functions as a pH-responsive carrier that securely retains HU under bloodstream conditions while causing release in an endosomal/acidic tumor environment. In contrast, CP−2 behaves as a less pH-sensitive host, offering weaker binding at pH 7.4, thus showing release of the drug in the blood stream and also affecting non-cancerous tissues. The targeted release of HU at cancerous cells is pronounced for CP−1, showing it to be a better drug carrier as compared to CP−2.

These findings are contraindicated with our previous hypothesis, suggesting CP−1 to be a better drug carrier and that targeted drug delivery is achieved better with CP−1 as compared to CP−2. Though azpy provides larger π-surface area than bpe, the N-N bond in between the two pyridyl moieties causes more electronegative repulsions as compared to the sp^2^ hybridized C-C bond, which causes weaker binding with the HU in the azpy.

### 4.3. Conformational Study Resolved MD Snapshots: Geometric Corroboration

**Figure 6:**
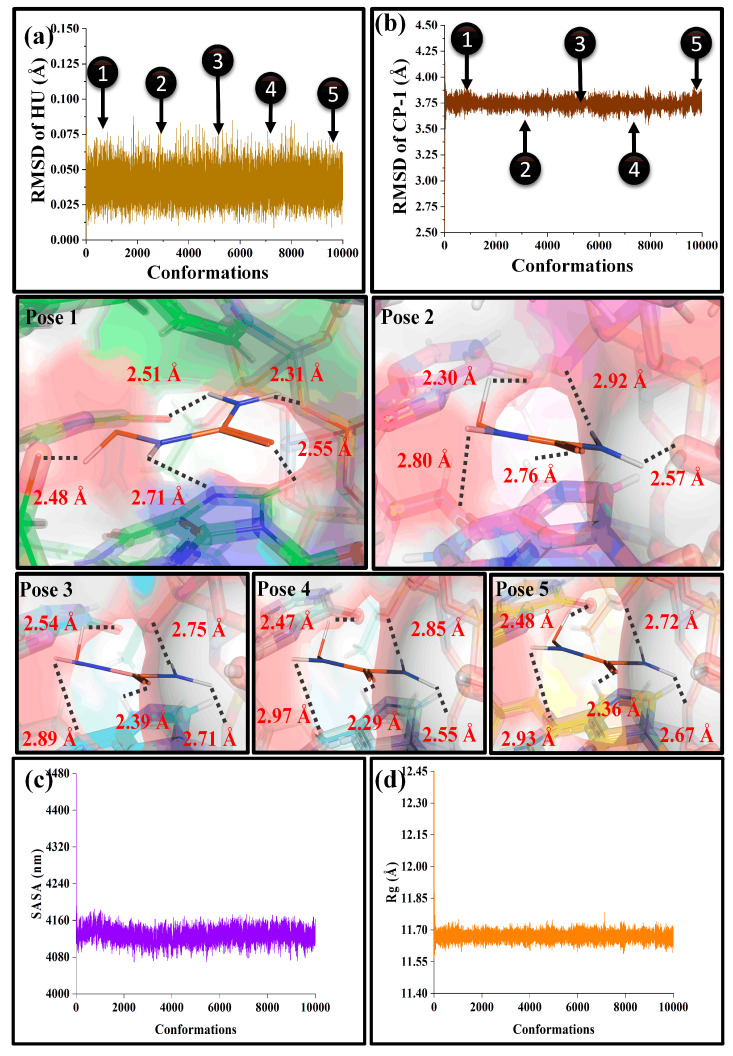
Molecular dynamics (MD) simulation of hydroxyurea binding to the CP−1 coordination polymer. (**a**) The HU graph of the RMSD versus conformations shows very minute fluctuation (0–0.15 Å). RMSD graph is also indicated by 1–5 numbered circles which indicate the five pose segments taken at (at 1000, 3000, 5000, 7000 and 9000 frames). (**b**) RMSD graph of CP−1 shows modest breathing between (2.5–4.5 Å) and the different pointers (1 to 5) show varying poses at different time intervals. The snapshots of the poses illustrate different binding capacities of HU with CP−1 at varying frames. (**c**) Solvent-accessible surface area (SASA), which is in the stable range throughout the simulation. (**d**) A steady radius of gyration indicates global compactness that is preserved throughout the computation, which indicates that the CP−1 does not undergo un-stability at any point; thus, the CP−1 is not releasing HU at blood pH (7.4) conditions.

**Figure 7:**
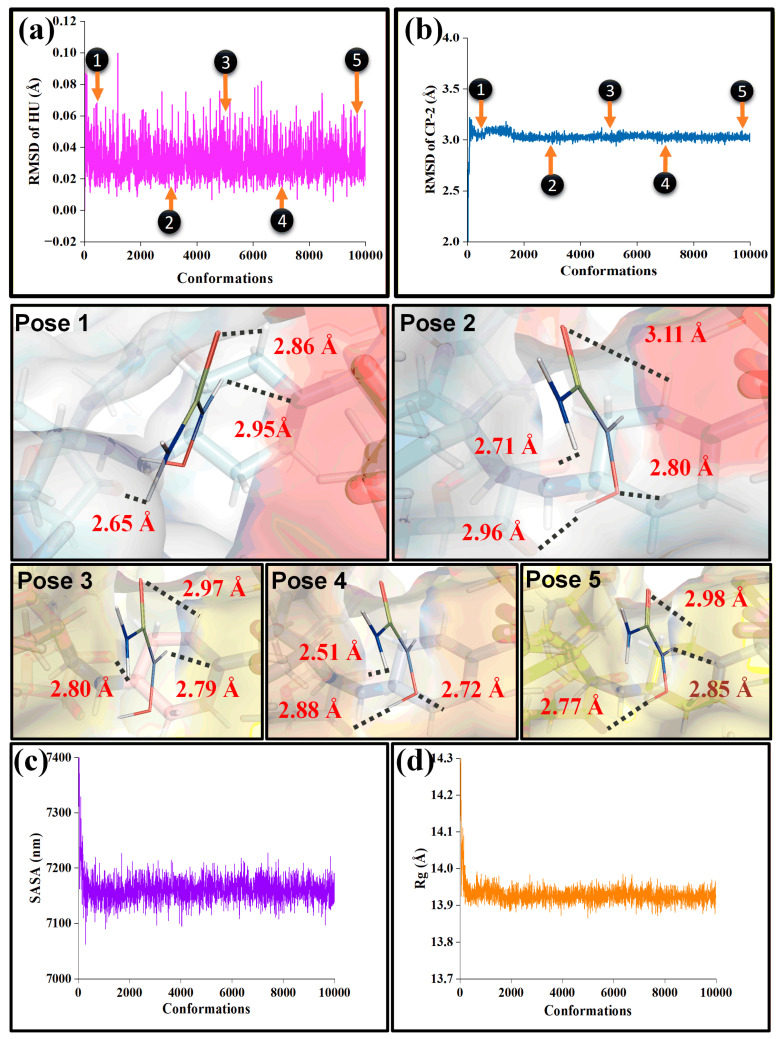
MD simulation analysis of HU drug binding with the CP−2 in five poses. (**a**) The RMSD graph of HU versus conformations indicates a small fluctuation and points out the 5 poses (at 1000, 3000, 5000, 7000 and 9000 frames) to check the structural inspection. (**b**) RMSD graph of CP−2 against the conformations shows the intermediate breathing and marks the 5 different poses like (**a**). The pictures of pose 1–5 indicate the interactions of CP−2 with the HU drug with dotted lines. (**c**) Solvent-accessible surface area (SASA) shows stability after equilibration and remains constant. (**d**) Radius of gyration (Rg) represents the moderate compactness of the CP−2 with the drug at blood pH 7.4 due to some spikes and weak band.

Five representative frames per system trace how docking-suggested motifs persist in dynamics. CP−1 repeatedly shows ≥3 “strong” contacts (≤2.70 Å) within each snapshot with typical sets such as 2.31/2.48/2.51/2.55/2.71 Å or 2.29/2.47/2.55/2.85/2.97 Å locating HU axially along the bpe tunnel and flanked by convergent pyridyl/IMP donors and acceptors (Figure 6). CP−2 places HU within the azpy channel but in flatter surface-biased orientations, with contact sets typically around 2.7–3.1 Å (2.71/2.80/2.96/3.11 Å), consistent with the larger Rg/SASA and the less favorable U (Figure 7). Thus, snapshots link the static docking cooperativity to dynamic retention, explaining why CP−1 sustains tighter binding, especially at neutral to basic pH.

### 4.4. Structure–Property Function Integration and Implications for Targeted Drug Delivery

The combined evidence from SCXRD verified chiral architectures, UV–vis fingerprints that separate and couple nucleotide or linker transitions, solid-state CD that registers M-type helicity, docking that quantifies multipoint contact cooperativity and MD that validates dynamic retention leads to a unified conclusion. CP−1 (IMP–Zn–bpe) is the superior hydroxyurea carrier. The difference in ΔG ≈ −3.28 kcalmol^−1^ predicts an ≈250-fold affinity advantage for CP−1 at 298 K. Dynamically at pH 7.4, the CP−1 pocket is more compact and more stabilizing (U lower by ~1700 kcalmol^−1^); while immobilizing HU to the same extent as CP−2 across pH, CP−1 retains HU most strongly at pH 9 ≥ 7.4 ≫ 5. Mechanistically, the bpe spacer provides conformational “give”, letting pyridyl vectors converge on the HU compact donor/acceptor triad to assemble several moderate-strength H-bonds at once. Moreover, the azpy planar electron-withdrawing azo unit is more rigid, reducing the probability of such multipoint convergence, and thereby biasing CP−2 toward faster release. Apart from that, the electrostatic repulsion due to N-N bonds in azpy with the N of HU also causes destabilization and thus, easy release of the drug. CP−1 can thus be a better targeted drug delivery carrier than CP−2. The future prospects of the current study also suggest to use both linker spacers of CP−1 and CP−2 in one assembly, harnessing the combined effect of load-in and sustained carriage offered by bpe from azpy. The combination ratios can therefore deploy controlled drug release at the targeted site.

## 5. Conclusions

This study advances Zn-IMP-based 3D coordination polymers (CP−1 and CP−2) as experimentally validated; computational modeling suggested these polymers are rationalized carriers for HU. Single-crystal X-ray diffraction confirms helical, permanently chiral three-dimensional networks crystallizing in the non-centrosymmetric space group *P2*_1_. The frameworks are built from Zn–O–N nodes that transmit axial chirality along phosphate–zinc chains, bridged by bpe or azpy ligands. MD integrates the environment with energetics at pH 7.4, CP−1 maintains a compact pore (lower Rg/SASA) yet yields far more favorable guest framework interaction energy than CP−2 and at pH 5, CP−1 maximizes HU immobilization and reduces interaction strength. Together, these convergent data reveal a clear structure–property relationship. Ethylene-linked bpe imparts local vector flexibility that enables multipoint convergence and deep-pocket binding whereas planar azpy enforces wall-aligned, less cooperative modes suited to faster release. Theoretically, CP−1 is the preferred platform for high-capacity loading and sustained carriage at pH greater than 7 while releasing the drug at pH 5, which suggests targeted cancer cell drug delivery. For CP−2, a more complementary release-prone architecture has been found at every pH; thus, the drug delivery is not targeted. Looking forward, explicit-solvent MD with MM/GBSA or FEP could refine free-energy partitions and buffer-controlled CD/UV–vis titrations could deliver apparent K_d_ and stoichiometry in vitro. More broadly, the design rules established here that linker-encoded axial chirality, cooperative H-bond topology and pH-tunable dynamics offer a scalable blueprint for nucleotide coordination frameworks that couple guest recognition to optical readouts and targeted drug delivery.

## Figures and Tables

**Figure 2:**
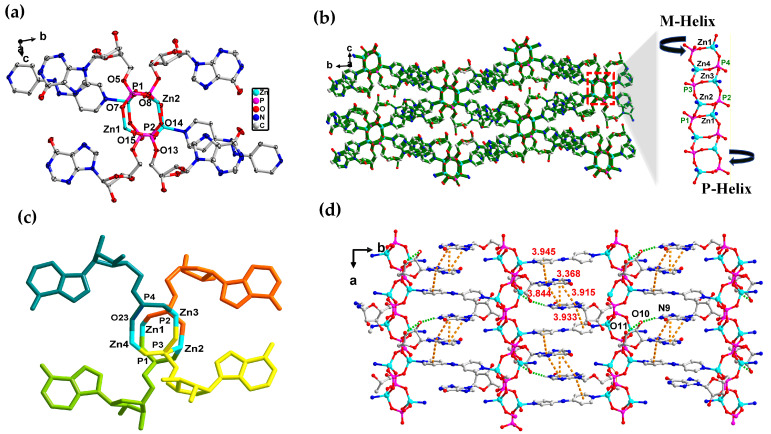
(**a**) Coordination environment of the Zn2, Zn4 center atoms and π-π stacking interactions in CP−2. (**b**) Coordination environment of the Zn1-Zn4 cluster of CP−2 connects with the phosphate atom of the IMP (H and auxiliary ligands are removed due to clarity). Hydrogen atoms and uncoordinated water molecules are removed for clarity. (**c**) Schematic presentation of the 3D architecture of CP−2 down-viewed from a-axis, and perspective view of the axial coordination and bridging mode of oxygen atom of the phosphate group and the central Zn(II) ion. (**d**) 3D coordination polymer of CP−2 formed by two different azpy ligands connecting the zinc–phosphate chains down-viewed from c-axis, as well as the abundant hydrogen bonds and multiple π-π stacking interactions in this 3D structure.

**Table 1:** Crystallographic data of CP−1 and CP−2.

	CP−1 {[Zn_4_(IMP)(bpe)_2_]·14H_2_O}n	CP−2 {[Zn_4_(IMP)(azpy)_2_]·14H_2_O}n
Empirical formula	C_64_P_4_O_46_Zn_4_N_20_H_92_	C_120_H_164_N_48_O_89_P_8_Zn_8_
Formula weight	2262.93	4473.70
Temperature/K	293(2)	296(2)
Crystal system	monoclinic	Monoclinic
Space group	*P2*_1_	*P2*_1_
a/Å	10.1405(5)	10.1689(7)
b/Å	30.6112(9)	29.778(2)
c/Å	15.1155(6)	15.1974(10)
α/°	90	90
β/°	108.523(5)	109.444(2)
γ/°	90	90
Volume/Å^3^	4449.0(3)	4339.4(5)
Z	2	1
ρ_calc_g/cm^3^	1.689	1.712
μ/mm^−1^	1.248	1.278
F(000)	2328.0	2292.0
Crystal size/mm^3^	0.23 × 0.18 × 0.12	0.34 × 0.18 × 0.17
Radiation	MoKα (λ = 0.71073)	MoKα (λ = 0.71073)
2Θ range for data collection/°	6.804 to 59.618	2.736 to 50.7
Index ranges	−13 ≤ h ≤ 14, −42 ≤ k ≤ 42, −20 ≤ l ≤ 20	−12 ≤ h ≤ 12, −35 ≤ k ≤ 35, −18 ≤ l ≤ 18
Reflections collected	51,930	43,468
Independent reflections	20603 [R_int_ = 0.0808, R_sigma_ = 0.0889]	15878 [R_int_ = 0.0272, R_sigma_ = 0.0439]
Data/restraints/parameters	20603/28/1243	15878/9/1237
Goodness-of-fit on F^2^	0.952	1.014
Final R indexes [I ≥ 2σ (I)]	R_1_ = 0.0552, wR_2_ = 0.1439	R_1_ = 0.0286, wR_2_ = 0.0729
Final R indexes [all data]	R_1_ = 0.0672, wR_2_ = 0.1510	R_1_ = 0.0318, wR_2_ = 0.0740
Largest diff. peak/hole / e Å^−3^	1.32/−1.40	0.93/−0.55
Flack parameter	0.024(9)	0.014(8)
CCDC no.	2504381	2504362

phosphate columns and stitches layer into a three-dimensional framework.

**Table 2:** Comparative table for the MD simulation studies of CP−1 and CP−2 with HU at different pH levels.

MD Simulations		RMSD Polymer		RMSD Drug		Rg		U (kcalmol^−1^)	
		CP−1 Å	CP−2	CP−1	CP−2	CP−1	CP−2	CP−1	CP−2
**pH**	5.0	3.46 ± 0.26	3.56 ± 0.15	0.34 ± 0.05	0.20 ± 0.02	11.73 ± 0.80	13.95 ± 0.06	−5992.17 ± 10.14	−4375.33 ± 215.19
	7.4	3.02 ± 0.11	3.74 ± 0.06	0.028 ± 0.01	0.038 ± 0.01	11.67 ± 0.03 Å	13.93 ± 0.03	−6062.18 ± 53.2	−4385.66 ± 95.50
	9.0	3.10 ± 0.29	3.38 ± 0.27	0.19 ± 0.04	0.26 ± 0.04	11.89 ± 0.14 Å	13.87 ± 0.07	−6029.17 ± 38.30	−4499.30 ± 214.10

## Data Availability

The original contributions presented in this study are included in the article. Further inquiries can be directed to the corresponding author.

## Supplementary Materials

The following supporting information can be downloaded at: https://www.mdpi.com/article/10.3390/polym18010119/s1.

## Abbreviations

The following abbreviations are used in this manuscript:

CP	Coordination Polymer
HU	Hydroxyurea (Drug)
MD	Molecular Dynamics
CD	Circular dichroism
IMP	Inosine monophosphate
bpe	1,2-bis(4-pyridine)ethylene
azpy	4,4′-azopyridine
XRD	X-ray diffraction
PXRD	Powder X-ray diffraction
U	Potential energy
K	Kinetic energy
SASA	Solvent-accessible surface area
Rg	Radius of gyration
H	Enthalpy
RMSD	Root mean square deviation
FTIR	Fourier transform infrared spectroscopy
UV–vis	UV–visible
FEP	Free Energy Perturbation
MM	Molecular Mechanics
PBSA	Poisson–Boltzmann Surface Area
