# Supplementary material for: Zn–IMP 3D Coordination Polymers for Drug Delivery: Crystal Structure and Computational Studies
Source: Polymers (Basel). 2025 Dec 31;18(1):119. doi: 10.3390/polym18010119 (PMC12787693; doi:10.3390/polym18010119)
Supplement: Supplementary file 1 [file polymers-18-00119-s001.zip › polymers-4036358-supplementary.pdf]

Supporting Information

Zn–IMP 3D Coordination Polymers for Drug Delivery: Crystal Structure and Computational Studies

Hafiz Zeshan Aqil [*1], Yanhong Zhu [*2], Masooma Hyder Khan [1], Yaqoot Khan [1], Beenish Sandhu [3], Muhammad Irfan [1] and Hui Li [1**]**

1 key Laboratory of Cluster of Science of Ministry of Education, School of Chemistry and Chemical Engineering Beijing Institute of Technology, Beijing 100081, P.R. China; hafizzeeshanaqil@gmail.com, yaqootkhan92@yahoo.com, Masoomakalyani@gmail.com
2 North China University of Science and Technology, School of Pharmacy 21 Bohai Road, Caofeidian Xincheng, Tangshan, Hebei, 063210, P.R. China; zhuyh@ncst.edu.cn
3 Department of Zoology, University of Sialkot Sialkot, 51040, Pakistan; benishsandhu17@gmail.com
*The equal contribution authors
** Hui Li; lihui@bit.edu.cn, Muhammad Irfan; irfanmuhammad@bit.edu.cn

1. Crystallographic Data and Structural Information

1.1. Selected Bond distances, Bond Angles, and Hydrogen Bonds of CP-1.

Table S1. Selected bond distances (Å) of CP-1.

**Atom**	**Atom**	**Length/Å**
Zn1	O1	1.927(5)
Zn1	O10	1.921(5)
Zn1	O27[1]	1.917(5)
Zn1	N20[2]	2.022(6)
Zn2	O3	1.930(5)
Zn2	O9	1.913(5)
Zn2	O18	1.908(5)
Zn2	N17	2.004(6)
Zn3	O11[3]	1.948(5)
Zn3	O19	1.898(5)
Zn3	O26	1.917(5)
Zn3	N18[4]	2.009(6)
Zn4	O2	1.925(5)
Zn4	O17	1.926(5)
Zn4	O25	1.932(5)
Zn4	N19	2.026(6)
P1	O17	1.517(6)
P1	O18	1.505(6)
P1	O19	1.508(5)
P1	O20	1.596(5)
P2	O9	1.513(6)
P2	O10	1.497(5)
P2	O11	1.510(6)
P2	O12	1.595(5)
P3	O25	1.525(5)
P3	O26	1.520(5)
P3	O27	1.509(5)
P3	O28	1.600(5)
P4	O1	1.512(5)
P4	O2	1.506(5)
P4	O3	1.522(5)
P4	O4	1.581(5)

[1]-1+X,+Y,+Z; [2]1-X,-1/2+Y,2-Z; [3]1+X,+Y,+Z; [4]1-X,-1/2+Y,1-Z

1.2. Table S2. Selected bond angles (°) of CP-1.

Atom	Atom	Atom	Angle/°	Atom	Atom	Atom	Angle/°
O1	Zn1	N20[1]	100.5(2)	O27	P3	O26	115.3(3)
O10	Zn1	O1	116.6(2)	O27	P3	O28	108.8(3)
O10	Zn1	N20[1]	106.0(2)	O1	P4	O3	112.6(3)
O27[2]	Zn1	O1	109.4(2)	O1	P4	O4	109.9(3)
O27[2]	Zn1	O10	111.5(2)	O2	P4	O1	108.6(3)
O27[2]	Zn1	N20[1]	112.4(3)	O2	P4	O3	115.7(3)
O3	Zn2	N17	104.1(3)	O2	P4	O4	109.1(3)
O9	Zn2	O3	112.8(2)	O3	P4	O4	100.5(3)
O9	Zn2	N17	109.1(3)	P4	O1	Zn1	131.3(3)
O18	Zn2	O3	116.1(2)	P4	O2	Zn4	133.7(4)
O18	Zn2	O9	105.5(2)	P4	O3	Zn2	126.4(3)
O18	Zn2	N17	109.1(3)	P2	O9	Zn2	133.0(4)
O11[3]	Zn3	N18[4]	107.2(3)	P2	O10	Zn1	136.9(4)
O19	Zn3	O11[3]	102.5(3)	P2	O11	Zn3[2]	122.5(3)
O19	Zn3	O26	112.3(2)	P1	O17	Zn4	139.9(4)
O19	Zn3	N18[4]	108.7(3)	P1	O18	Zn2	128.0(3)
O26	Zn3	O11[3]	123.5(2)	P1	O19	Zn3	135.4(4)
O26	Zn3	N18[4]	102.0(2)	P3	O25	Zn4	127.6(3)
O2	Zn4	O17	116.0(2)	P3	O26	Zn3	142.9(3)
O2	Zn4	O25	110.8(2)	P3	O27	Zn1[3]	133.2(4)
O2	Zn4	N19	106.3(2)	O9	P2	O12	110.5(3)
O17	Zn4	O25	110.2(2)	O10	P2	O9	115.7(3)
O17	Zn4	N19	106.3(3)	O10	P2	O11	111.3(3)
O25	Zn4	N19	106.7(2)	O10	P2	O12	102.4(3)
O17	P1	O20	101.7(3)	O11	P2	O9	109.6(3)
O18	P1	O17	113.5(3)	O11	P2	O12	106.8(3)
O18	P1	O19	108.9(3)	O25	P3	O28	107.9(3)
O18	P1	O20	108.3(4)	O26	P3	O25	113.8(3)
O19	P1	O17	115.9(3)	O26	P3	O28	101.0(3)
O19	P1	O20	107.9(3)	O27	P3	O25	109.4(3)

[1]1-X,-1/2+Y,2-Z; [2]-1+X,+Y,+Z; [3]1+X,+Y,+Z; [4]1-X,-1/2+Y,1-Z; [5]1-X,1/2+Y,1-Z; [6]1-X,1/2+Y,2-Z

1.3. Table S3. Selected H-bonding distances (Å) and angles (°) of CP-1.

D	H	A	d(D-H)/Å	d(H-A)/Å	d(D-A)/Å	D-H-A/°
O6	H6C	O44	0.82	2.07	2.886(9)	173.1
O7	H7C	O39[1]	0.82	2.07	2.874(10)	167.8
O14	H14C	O15	0.82	2.30	2.777(9)	118.0
O15	H15D	O44[2]	0.82	2.18	2.940(9)	154.3
O22	H22C	O11[3]	0.82	2.06	2.869(8)	168.7
O23	H23C	O46	0.82	1.94	2.754(10)	170.4
O30	H30C	O31	0.82	2.38	2.797(9)	112.4
O30	H30C	O44[3]	0.82	2.47	3.173(8)	145.1
O31	H31E	O24[4]	0.82	1.87	2.686(9)	176.6
O34	H34B	O5[2]	0.85	2.49	2.896(10)	110.2
O34	H34C	O14	0.85	2.17	2.648(11)	115.1
O35	H35C	O29[2]	0.85	2.60	3.212(16)	129.6
O35	H35C	O40[2]	0.85	2.33	3.06(2)	144.8
O35	H35D	O15	0.85	2.10	2.834(13)	144.5
O36	H36C	O37	0.85	2.01	2.782(14)	150.9
O36	H36D	O38	0.85	1.95	2.73(3)	151.0
O37	H37C	O3[5]	0.85	2.18	3.027(10)	174.0
O37	H37D	O25[6]	0.85	2.09	2.937(10)	174.1
O38	H38C	O9[5]	0.85	2.10	2.94(2)	167.6
O38	H38D	O17[6]	0.85	2.17	3.00(2)	167.2
O39	H39C	O21[6]	0.85	1.93	2.777(9)	174.6
O39	H39D	O32	0.85	1.88	2.727(10)	174.5
O41	H41C	O1	0.85	2.03	2.861(9)	167.5
O41	H41D	O26	0.85	2.09	2.922(9)	167.6
O42	H42B	O18	0.85	2.50	3.169(12)	136.8
O42	H42B	O19	0.85	2.63	3.357(12)	143.7
O43	H43C	O13	0.85	2.25	2.771(8)	119.5
O44	H44B	O43[7]	0.85	2.36	2.797(9)	112.5
O45	H45C	O8	0.85	1.92	2.709(15)	154.1
O45	H45D	O24	0.85	2.06	2.848(14)	154.3
O46	H46C	O16[8]	0.85	1.91	2.754(9)	170.2
O46	H46D	O32[9]	0.85	2.12	2.965(9)	170.5
N4	H4A	O40[1]	0.86	1.94	2.781(16)	166.4
N8	H8	O43[8]	0.86	1.96	2.817(9)	171.2
N11	H11	O7[10]	0.86	2.20	3.038(9)	165.9
N15	H15A	O36	0.86	1.99	2.769(13)	149.8

[1]1-X,1/2+Y,2-Z; [2]-1+X,+Y,-1+Z; [3]1+X,+Y,+Z; [4]2-X,-1/2+Y,2-Z; [5]-X,-1/2+Y,1-Z; [6]1-X,-1/2+Y,1-Z; [7]+X,+Y,1+Z; [8]1-X,1/2+Y,1-Z; [9]2-X,1/2+Y,1-Z; [10]1-X,-1/2+Y,2-Z

2. Selected Bond distances, Bond Angles, and Hydrogen Bonds of CP-2.

2.1. Table S4. Selected bond distances (Å) of CP-2.

Atom	Atom	Length/Å	Atom	Atom	Length/Å
Zn1	O7	1.918(3)	N8	C16	1.460(6)
Zn1	O15	1.930(3)	N9	C21	1.399(7)
Zn1	O22[1]	1.923(3)	N9	C22	1.350(6)
Zn1	N13[2]	2.025(4)	N10	C22	1.302(6)
Zn2	O8	1.928(3)	N10	C23	1.377(6)
Zn2	O14	1.928(3)	N11	C24	1.380(6)
Zn2	O32	1.911(3)	N11	C25	1.308(7)
Zn2	N17[3]	2.003(4)	N12	C23	1.372(6)
Zn3	O16	1.925(3)	N12	C25	1.386(6)
Zn3	O24	1.932(3)	N12	C26	1.468(6)
Zn3	O30	1.915(3)	N13	C31	1.330(6)
Zn3	N16	2.034(4)	N13	C33	1.348(6)
Zn4	O6[4]	1.942(3)	N14	N15	1.232(6)
Zn4	O23	1.915(3)	N14	C35	1.433(6)
Zn4	O31	1.907(4)	N15	C36	1.448(6)
Zn4	N20	2.023(4)	N16	C38	1.352(6)
P1	O5	1.596(4)	N16	C40	1.325(6)
P1	O6	1.517(4)	N17	C41	1.334(6)
P1	O7	1.505(4)	N17	C43	1.334(6)
P1	O8	1.504(3)	N18	N19	1.235(6)
P2	O13	1.587(3)	N18	C45	1.438(6)
P2	O14	1.522(3)	N19	C46	1.439(6)
P2	O15	1.519(3)	N20	C48	1.345(6)
P2	O16	1.506(3)	N20	C49	1.349(6)
P3	O29	1.598(3)	N21	C51	1.385(7)
P3	O30	1.509(3)	N21	C52	1.360(7)
P3	O31	1.493(4)	N22	C52	1.308(7)
P3	O32	1.506(4)	N22	C53	1.341(7)
P4	O21	1.592(3)	N23	C54	1.392(7)
P4	O22	1.513(4)	N23	C55	1.300(7)
P4	O23	1.518(3)	N24	C53	1.380(6)
P4	O24	1.522(3)	N24	C55	1.378(6)

[1]1+X,+Y,+Z; [2]1-X,-1/2+Y,1-Z; [3]1-X,1/2+Y,2-Z; [4]-1+X,+Y,+Z

2.2. Table S5. Selected bond angles (°) of CP-2.

Atom	Atom	Atom	Angle/°	Atom	Atom	Atom	Angle/°
O7	Zn1	O15	112.48(15)	O32	P3	O29	107.4(2)
O7	Zn1	O22[1]	113.69(15)	O32	P3	O30	113.4(2)
O7	Zn1	N13[2]	107.41(16)	O22	P4	O21	109.36(19)
O15	Zn1	N13[2]	101.18(15)	O22	P4	O23	114.1(2)
O22[1]	Zn1	O15	110.33(14)	O22	P4	O24	110.08(19)
O22[1]	Zn1	N13[2]	111.02(15)	O23	P4	O21	100.54(18)
O8	Zn2	N17[3]	108.87(16)	O23	P4	O24	113.89(19)
O14	Zn2	O8	112.38(14)	O24	P4	O21	108.21(18)
O14	Zn2	N17[3]	104.41(16)	P1	O6	Zn4[1]	123.1(2)
O32	Zn2	O8	104.52(14)	P1	O7	Zn1	137.3(2)
O32	Zn2	O14	117.53(15)	P1	O8	Zn2	131.0(2)
O32	Zn2	N17[3]	108.96(16)	P2	O14	Zn2	124.8(2)
O16	Zn3	O24	111.21(14)	P2	O15	Zn1	128.8(2)
O16	Zn3	N16	105.64(16)	P2	O16	Zn3	135.3(2)
O24	Zn3	N16	106.93(14)	P4	O22	Zn1[4]	131.2(2)
O30	Zn3	O16	113.71(15)	P4	O23	Zn4	141.7(2)
O30	Zn3	O24	111.54(14)	P4	O24	Zn3	129.56(19)
O30	Zn3	N16	107.32(16)	P3	O30	Zn3	140.1(2)
O6[4]	Zn4	N20	107.12(16)	P3	O31	Zn4	135.4(2)
O23	Zn4	O6[4]	122.94(15)	P3	O32	Zn2	126.4(2)
O23	Zn4	N20	102.06(15)	O14	P2	O13	101.52(18)
O31	Zn4	O6[4]	103.79(16)	O15	P2	O13	108.90(19)
O31	Zn4	O23	112.85(16)	O15	P2	O14	112.55(19)
O31	Zn4	N20	107.15(16)	O16	P2	O13	109.23(19)
O6	P1	O5	106.4(2)	O16	P2	O14	115.26(19)
O7	P1	O5	101.2(2)	O16	P2	O15	109.0(2)
O7	P1	O6	112.2(2)	O30	P3	O29	102.31(19)
O8	P1	O5	110.37(19)	O31	P3	O29	107.8(2)
O8	P1	O6	109.6(2)	O31	P3	O30	116.6(2)
O8	P1	O7	116.3(2)	O31	P3	O32	108.7(2)

[1]1+X,+Y,+Z; [2]1-X,-1/2+Y,1-Z; [3]1-X,1/2+Y,2-Z; [4]-1+X,+Y,+Z; [5]1-X,1/2+Y,1-Z; [6]1-X,-1/2+Y,2-Z

2.3. Table S6. Selected H-bonding distances (Å) and angles (°) of CP-2.

D	H	A	d(D-H)/Å	d(H-A)/Å	d(D-A)/Å	D-H-A/°
O2	H2	O42[1]	0.82	1.96	2.765(6)	166.8
O3	H3	O41[1]	0.82	2.00	2.721(6)	146.3
O10	H10	O11	0.82	2.30	2.704(5)	111.2
O10	H10	O37	0.82	2.19	2.842(5)	136.5
O11	H11	O34	0.82	2.04	2.847(5)	170.4
O18	H18	O25[2]	0.82	1.83	2.650(5)	176.1
O26	H26	O38[3]	0.82	1.95	2.736(6)	159.0
O27	H27	O6[4]	0.82	1.95	2.765(5)	172.3
O35	H35B	O31	0.85	2.62	3.279(6)	135.3
O35	H35B	O32	0.85	2.38	3.107(6)	143.8
O35	H35C	O46	0.85	2.33	3.110(15)	153.5
O39	H39C	O40[4]	0.85	2.43	2.835	110.2
O39	H39C	N17[5]	0.85	2.66	3.296(5)	132.3
O40	H40B	O22[6]	0.85	2.61	3.414(5)	159.1
O40	H40B	O24[6]	0.85	2.49	2.897(4)	110.6
O37	H37A	O17[7]	0.85	1.91	2.698(5)	154.7
O37	H37B	O28[8]	0.86	1.95	2.758(5)	156.6
O41	H41A	O36[4]	0.85	1.89	2.725(6)	167.5
O41	H41B	O12	0.85	2.15	2.846(5)	139.1
O42	H42C	O9[9]	0.85	2.14	2.972(6)	167.2
O42	H42D	O20	0.85	2.14	2.975(6)	167.2
O43	H43C	O15	0.85	2.00	2.836(7)	165.6
O43	H43C	O16	0.85	2.64	3.273(7)	132.0
O43	H43D	O23	0.85	2.07	2.904(7)	165.4
N1	H1	O39[10]	0.86	2.11	2.973(8)	178.3
N5	H5	O44[7]	0.86	1.88	2.721(8)	166.3
N5	H5	O46[7]	0.86	2.34	3.060(15)	141.5
N9	H9	O10[9]	0.86	2.06	2.903(5)	167.0
N21	H21	O33[7]	0.86	1.95	2.802(5)	170.7

[1]1+X,+Y,1+Z; [2]-X,-1/2+Y,1-Z; [3]+X,+Y,1+Z; [4]-1+X,+Y,+Z; [5]-X,1/2+Y,2-Z; [6]1+X,+Y,+Z; [7]1-X,1/2+Y,1-Z; [8]+X,+Y,-1+Z; [9]1-X,-1/2+Y,1-Z; [10]1-X,-1/2+Y,2-Z

3. Table S7. The calculated values of the phase angle of pseudorotation P and of the amplitude of Pucker τm of the pentose ring for the samples of CP-1 and CP-2.[1]

Compound	P /°	τm /°	τ2 /°	τ3 /°	τ4 /°	τ0 /°	τ1 /°	Conventional Notation	χCN
**CP-1**	177.84	37.94	37.909	30.728	–10.77	13.61	32.298	C(2')–endo $^2T_3$	118
	12.10	40.37	39.477	–37.51	21.325	4.659	–28.15	C(3')–endo 3E	149
	164.76	32.46	31.314	21.403	–2.026	18.32	31.364	C(2')–endo 2E	122
	12.72	38.03	37.093	-35.77	19.672	3.887	-25.78	C(3')–endo 3E	137
**CP-2**	13.17	42.61	41.493	–39.99	22.929	4.006	–29.03	C(3')–endo 3E	155
	167.07	32.85	-32.019	22.555	-3.438	-17.47	31.152	C(2')–endo 2E	-122
	9.38	38.15	37.637	–34.70	17.993	6.043	–27.52	C(3')–endo 3E	–136
	176.01	37.70	–37.609	29.671	–9.618	14.59	32.769	C(2')–endo $^2T_3$	–119

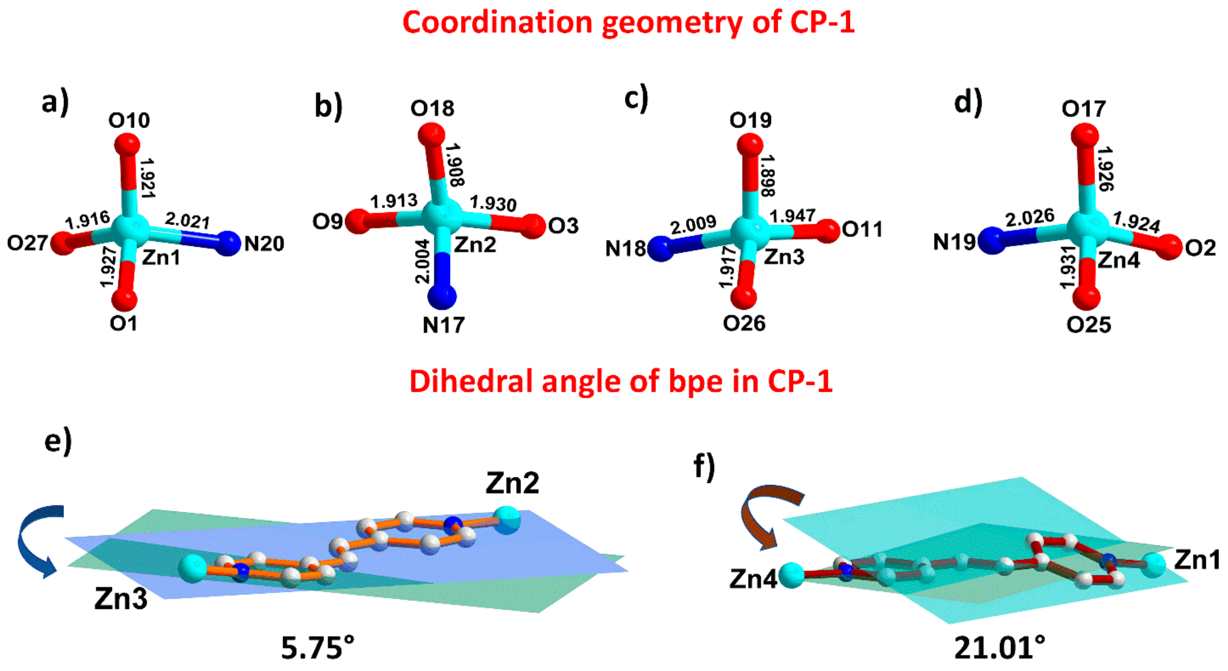

Figure S1. Coordination geometry and dihedral angle of auxiliary ligand bpe in CP-1.

“ Boat and Chair ” conformations

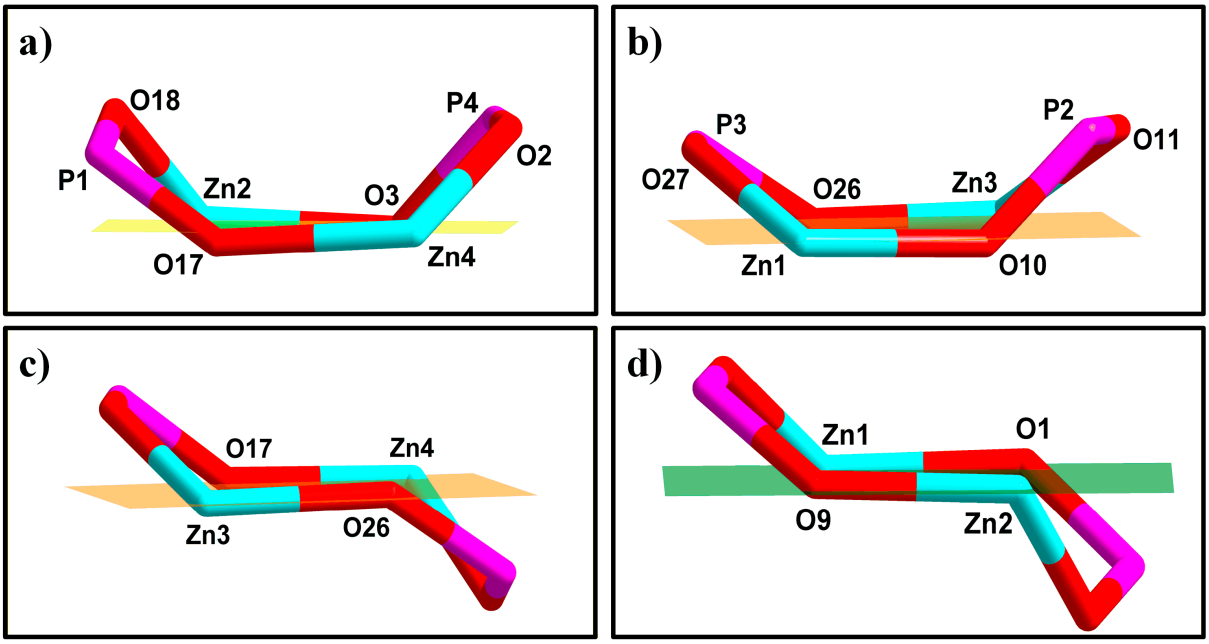

Figure S2. Boat and Chair conformation of CP-1.

“ Envelope and Twist ” conformations

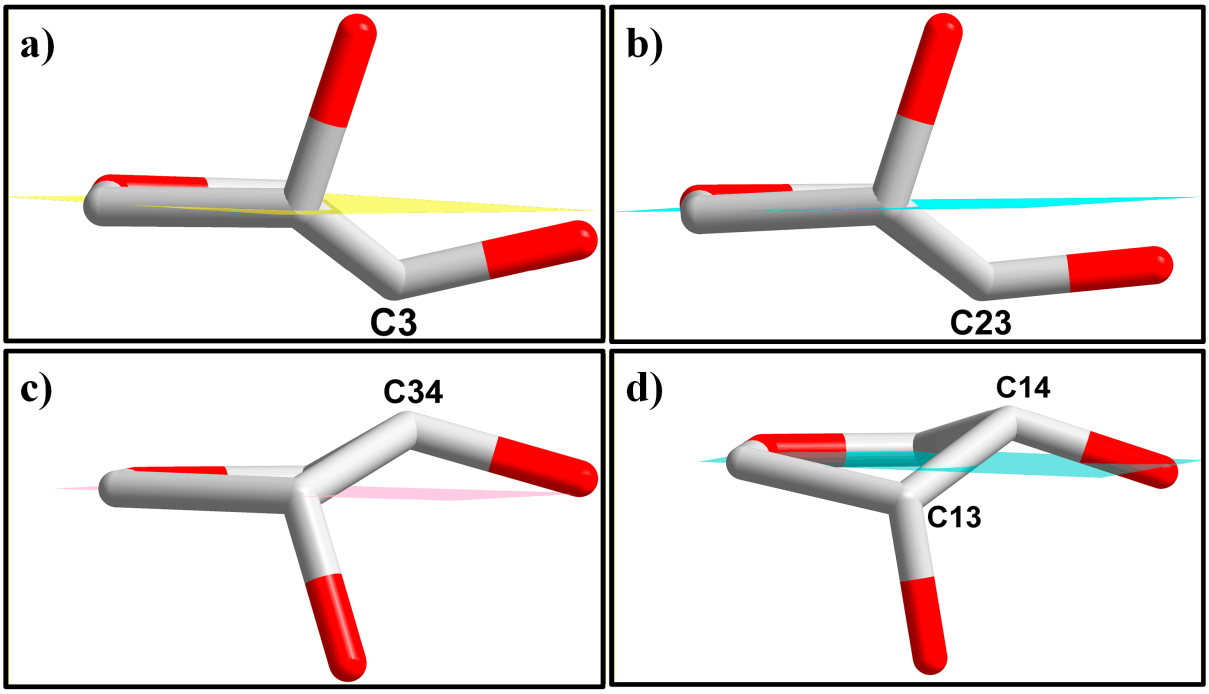

Figure S3. Envelope and Twist conformation of CP-1.

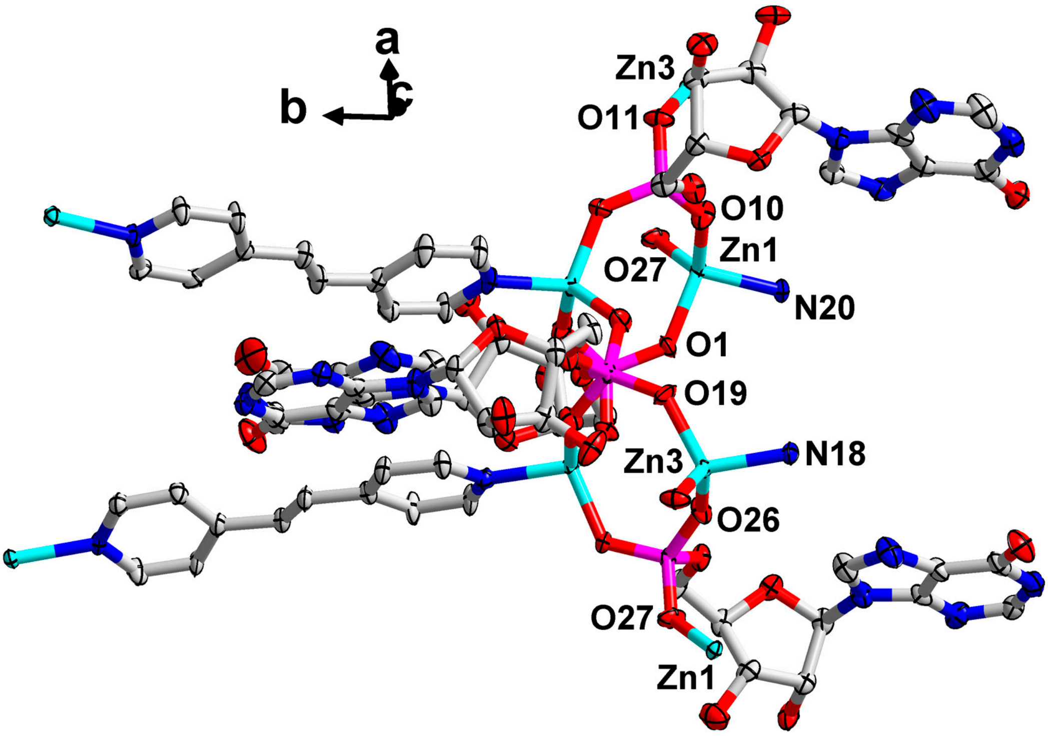

Figure S4. ORTEP picture of CP-1

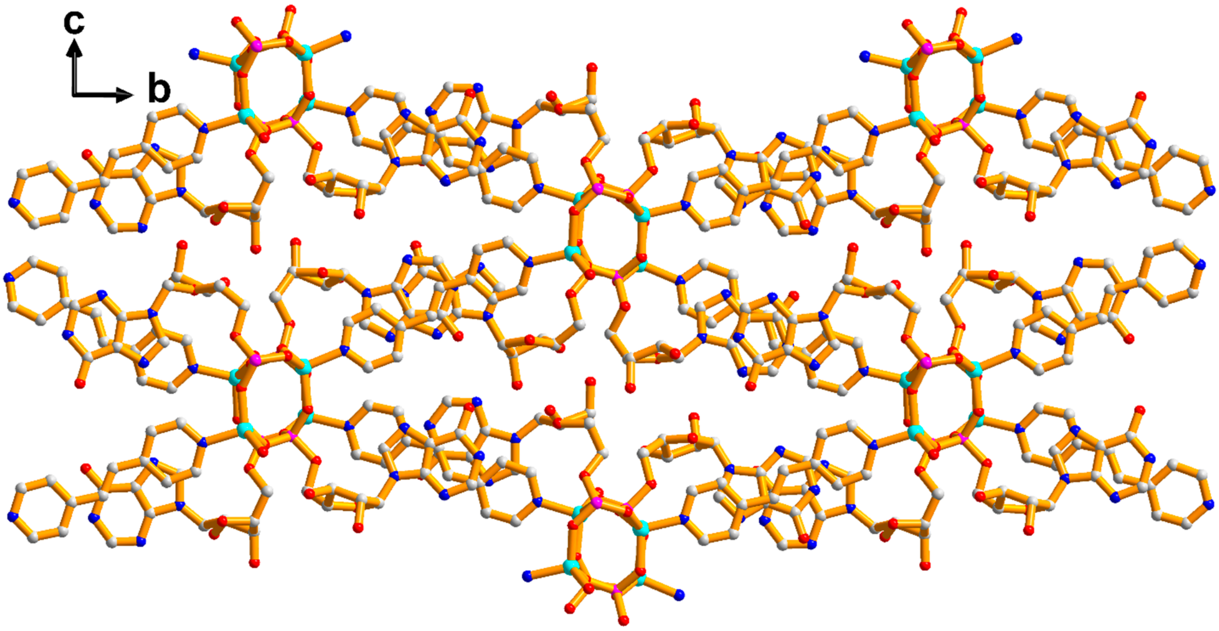

Figure S5. 3D structure of (CP-1) view along a-axis.

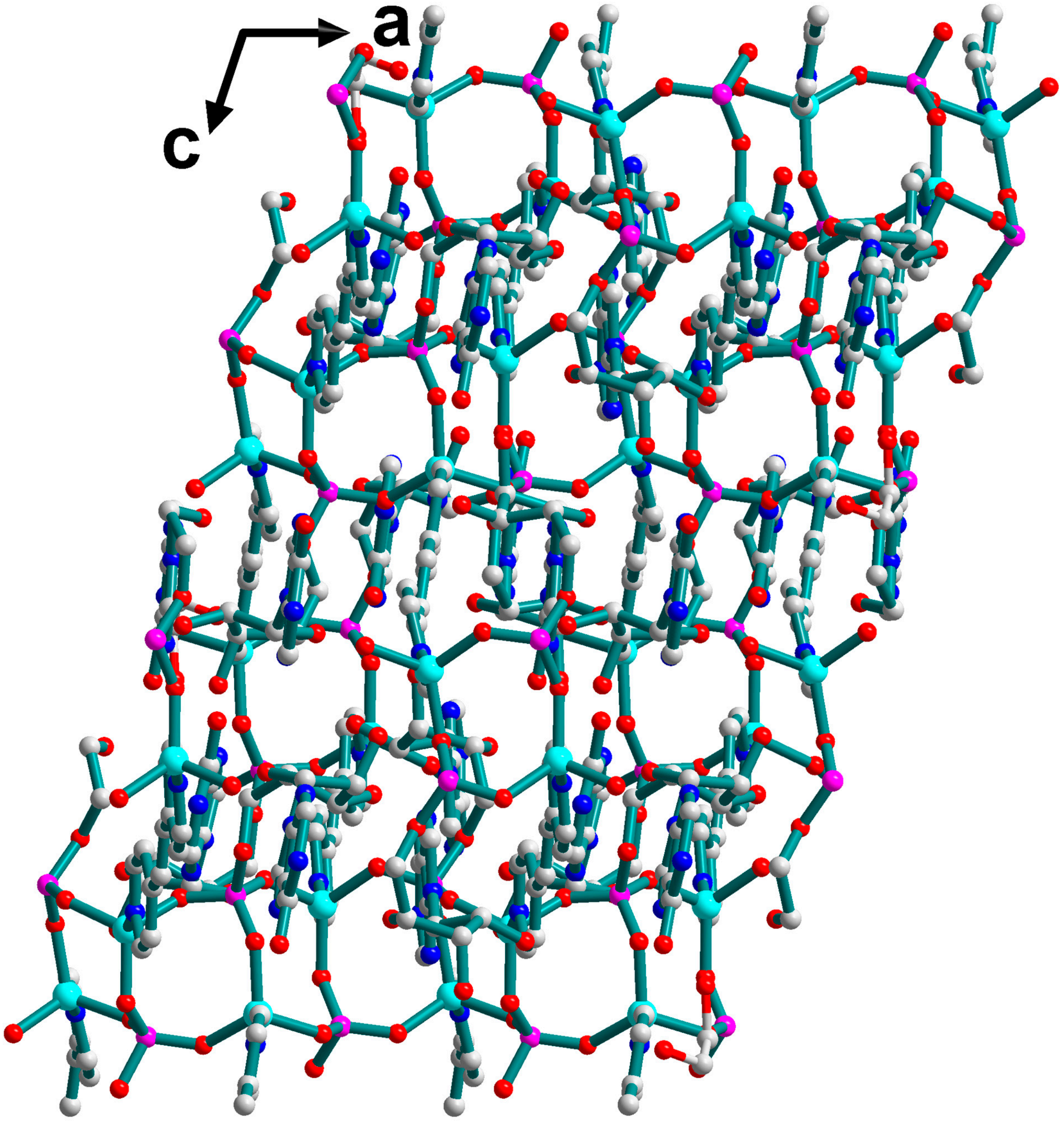

Figure S6. 3D structure of (CP-1) view along b-axis.

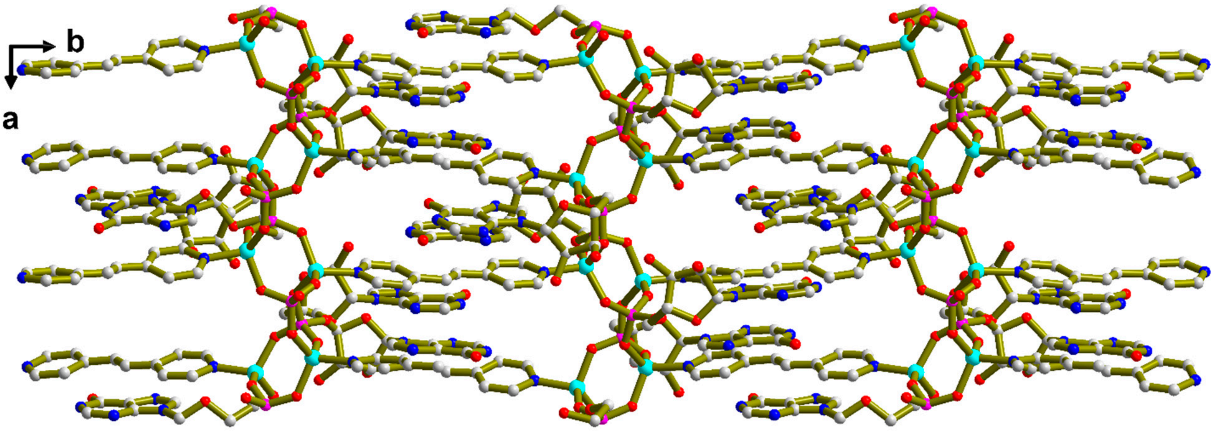

Figure S7. 3D structure of CP-1 view along c-axis.

Supporting information of CP-2

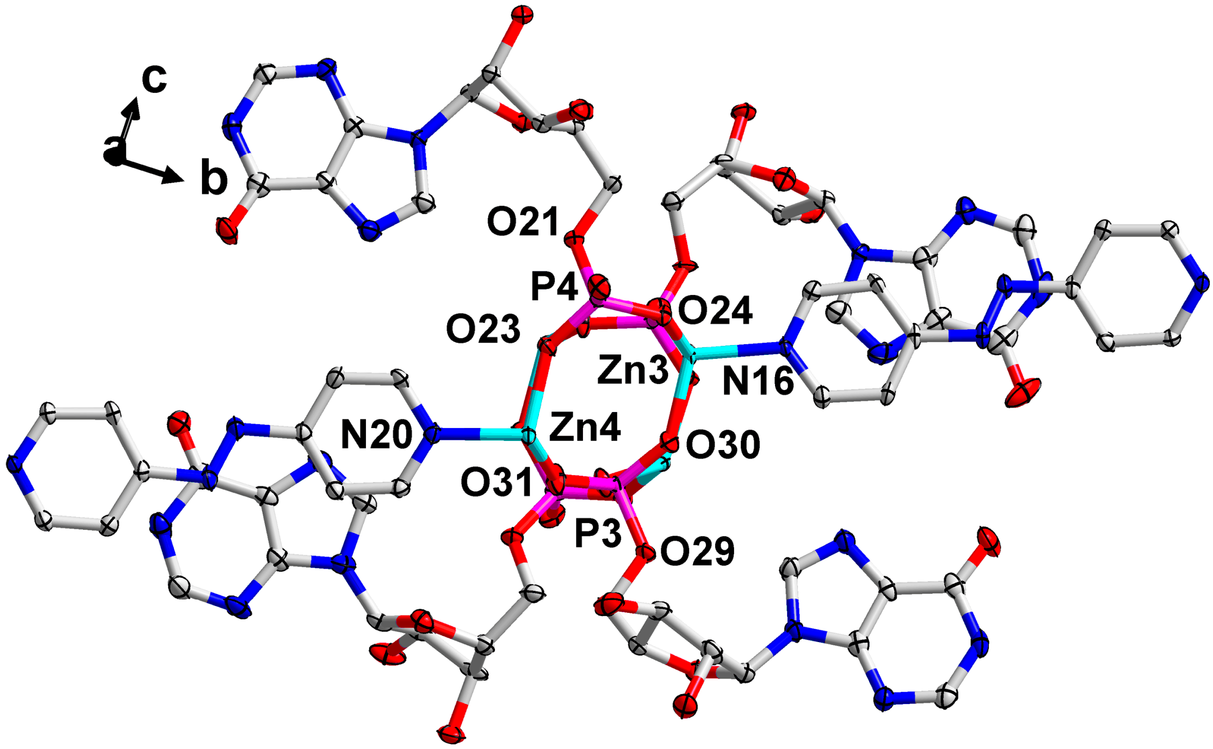

Figure S8. ORTEP picture of CP-2 (Hydrogen atoms and uncoordinated water molecules and are removed for clarity).

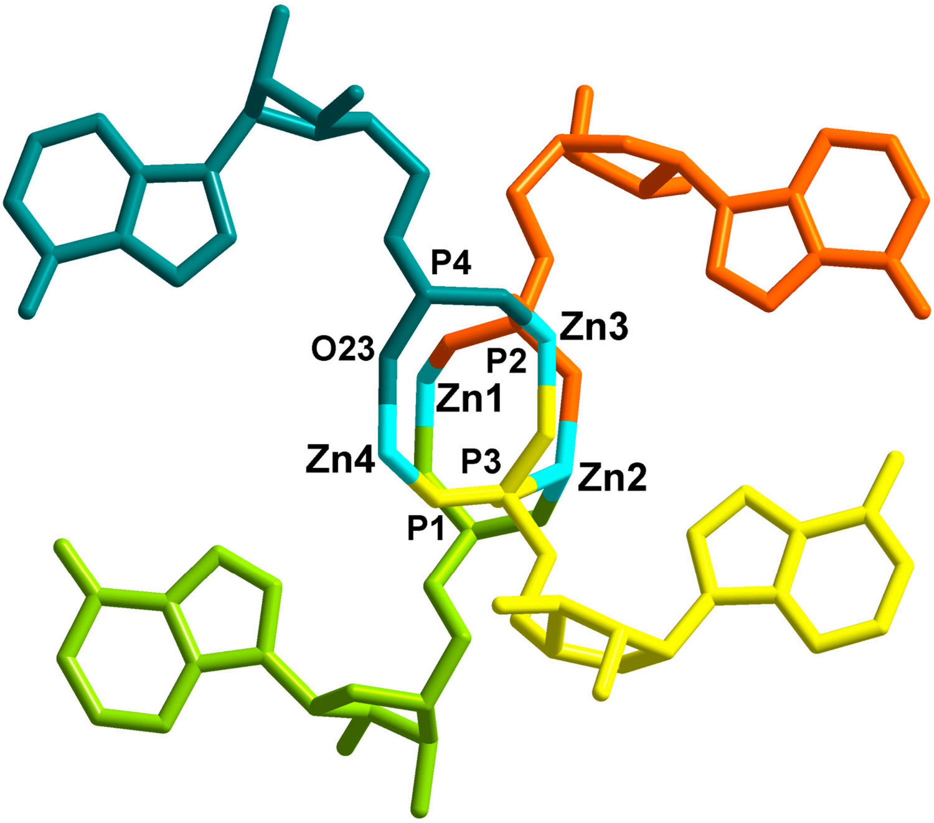

Figure S9. Coordination environment of the Zn1-Zn4 cluster of CP-2 connect with the phosphate atom of the IMP (H and auxiliary ligands are removed due to clarity). Hydrogen atoms and uncoordinated water molecules and are removed for clarity

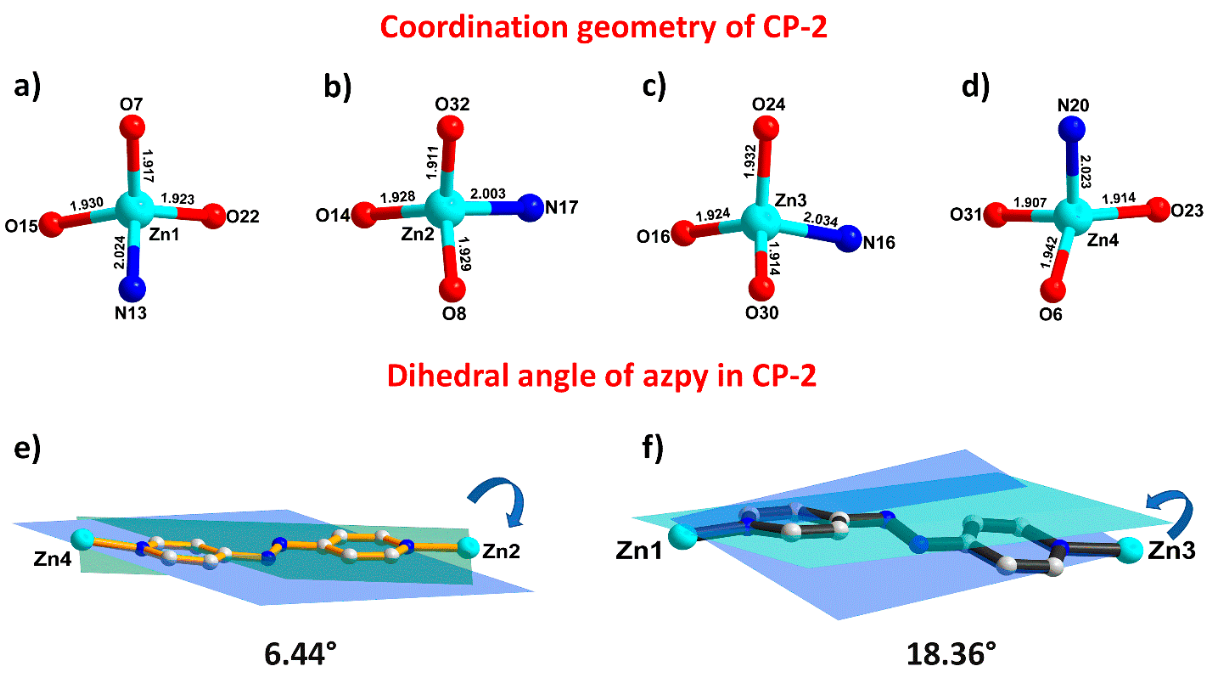

Figure S10. Coordination geometry and dihedral angle of auxiliary ligand azpy in CP-2.

“ Boat and Chair ” conformations

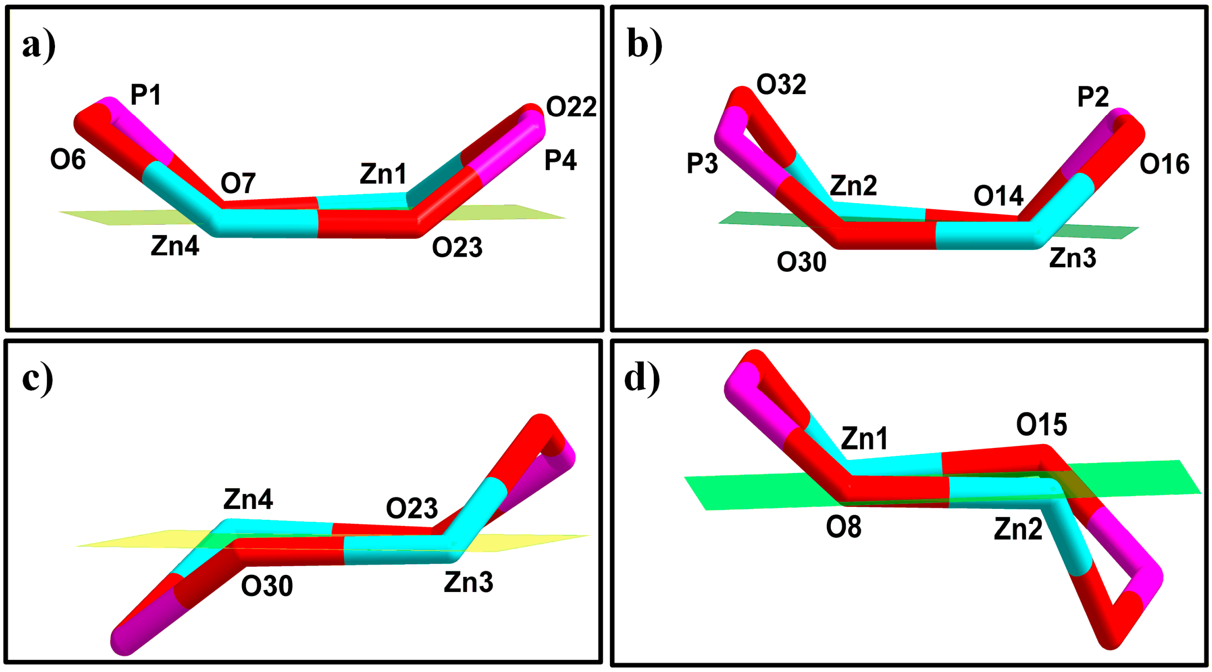

Figure S11. Boat and Chair conformation of CP-2.

“ Envelope and Twist ” conformations

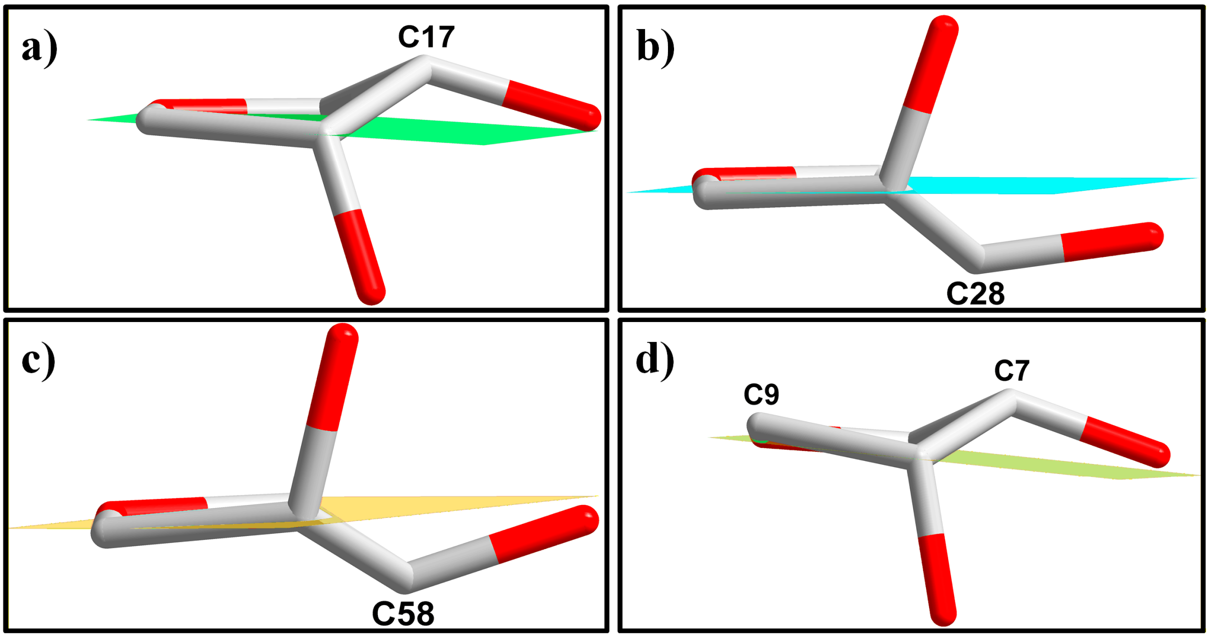

Figure S12. Envelope and Twist conformation of sugar ring of CP-2.

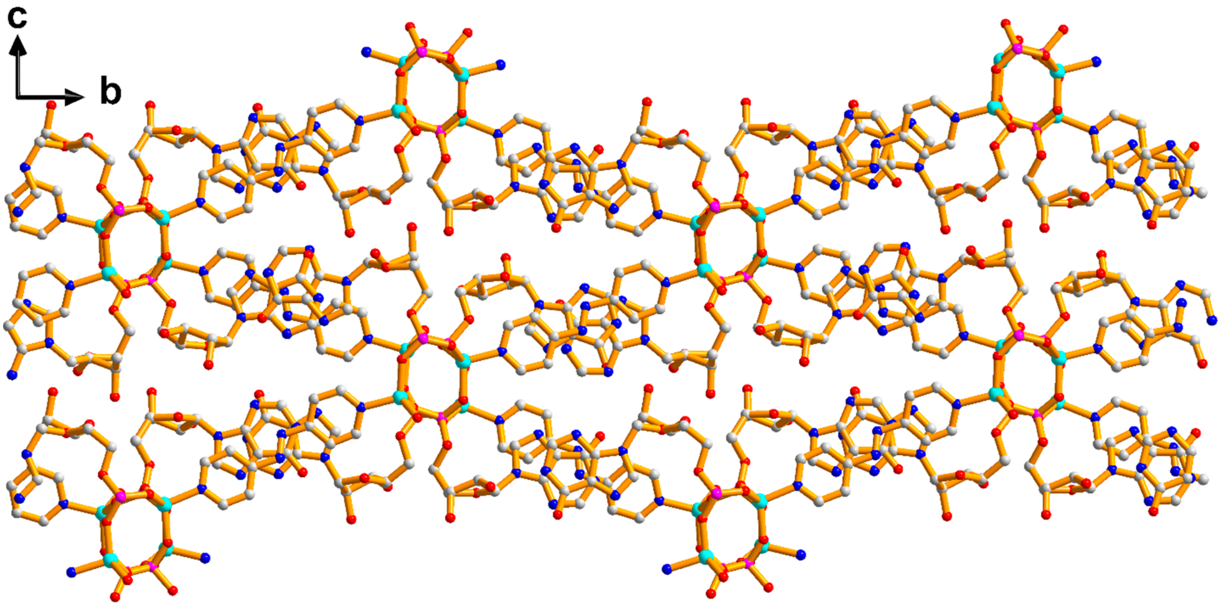

Figure S13. 3D structure of (CP-2) view along a-axis.

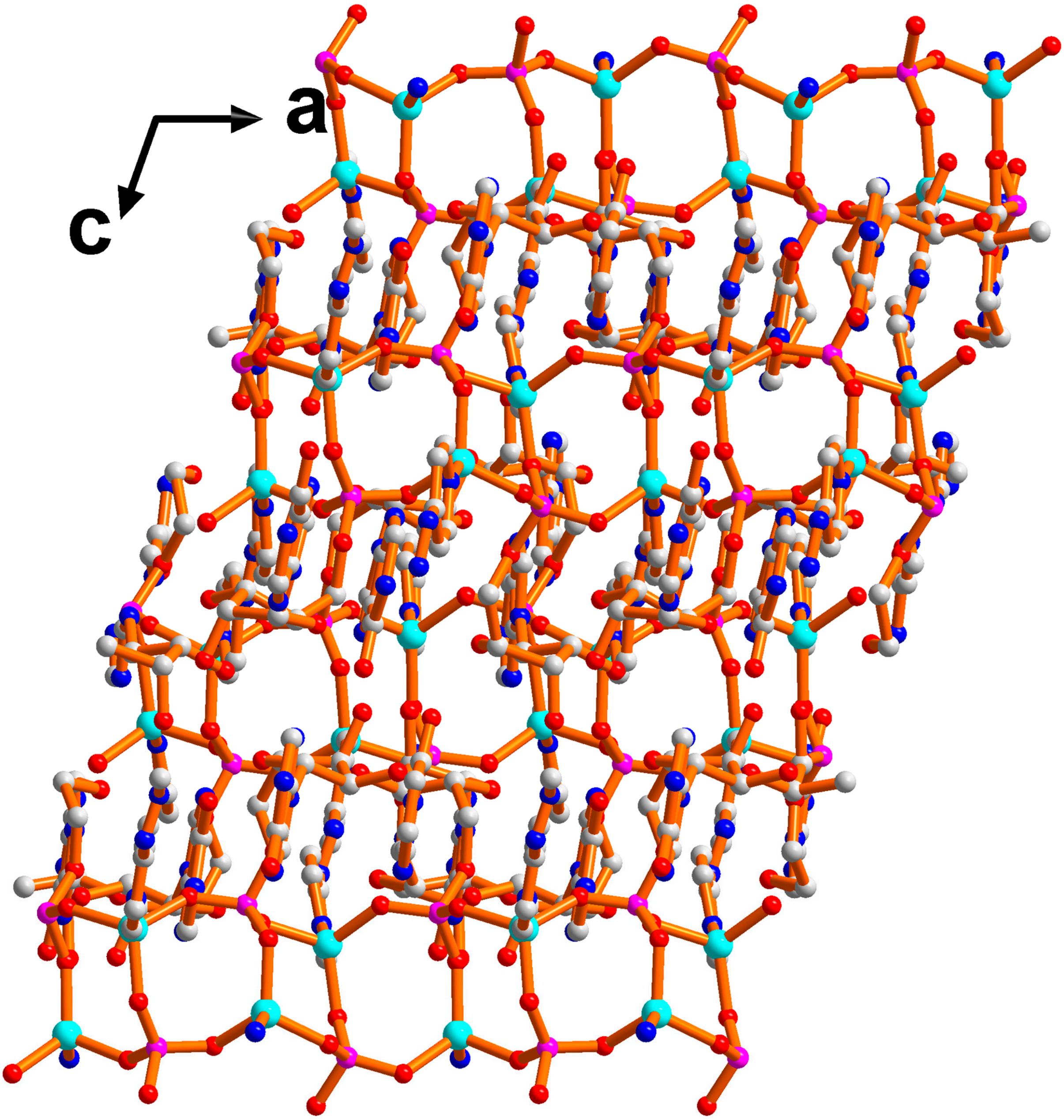

Figure S14. 3D structure of (CP-2) view along b-axis.

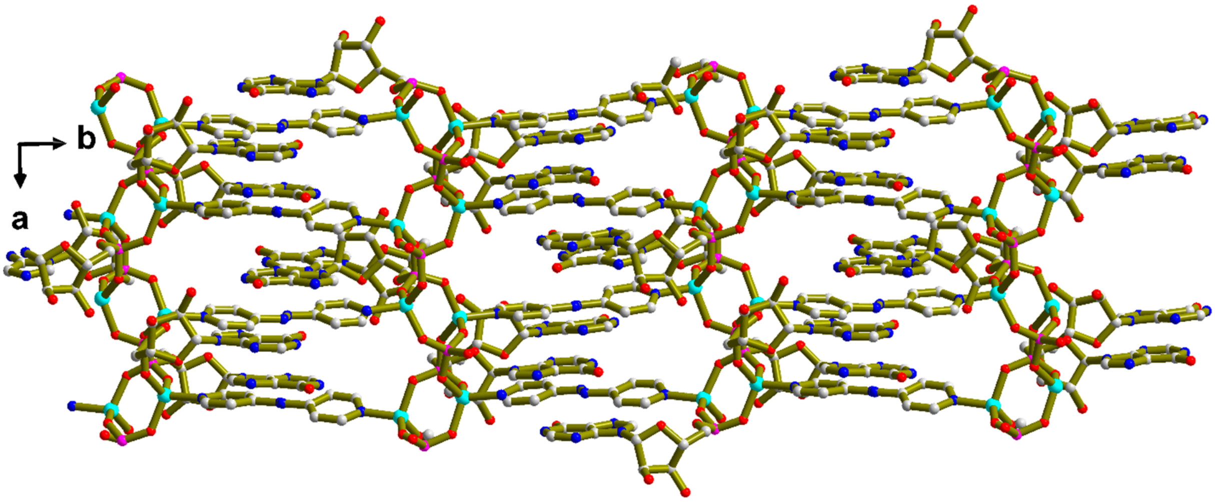

Figure S15. 3D structure of (CP-2) view along c-axis.

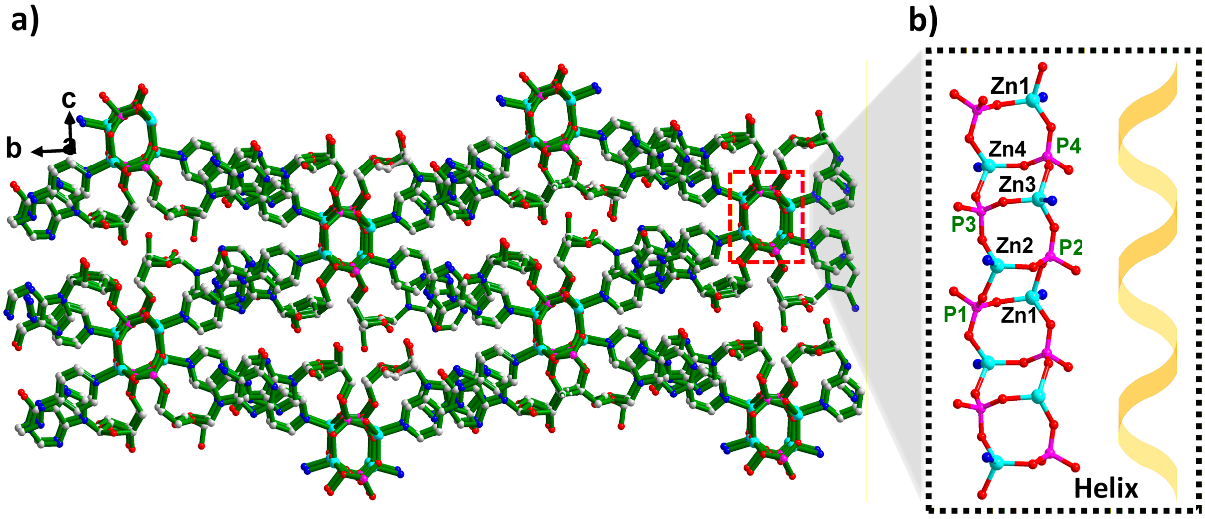

Figure S16. Schematic presentation of the 3D architecture of CP-2 down viewed from a-axis. (b) Perspective view of the coordination and bridging mode of oxygen atom of the phosphate group and the central Zn(II) ion.

The PXRD Patterns of CP-1 and CP-2

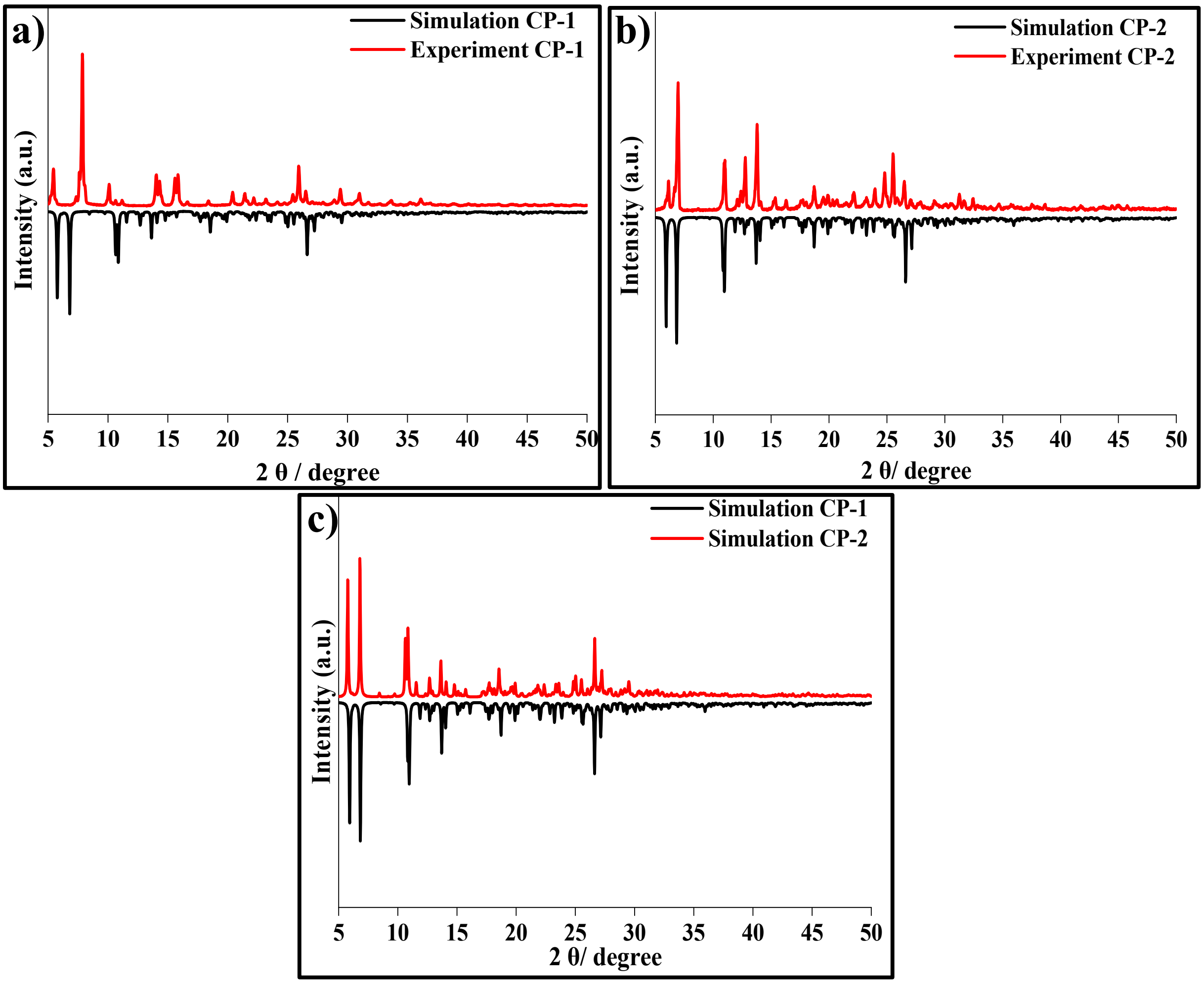

Figure S17. PXRD patterns show the comparison between the experimental values and calculated ones for (a) CP-1, (b) CP-2, (c) simulation of CP-1 and CP-2.

FTIR Spectroscopy of CP-1 and CP-2.

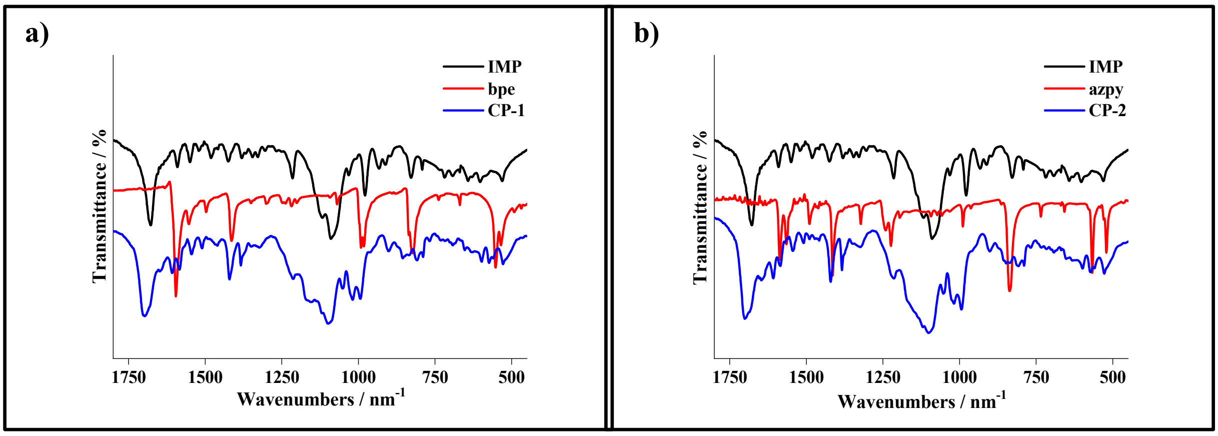

Figure S18. IR spectra of (a) IMP, bpe and CP-1; (b) IMP, azpy and CP-2.

The Solution-State UV-vis Spectra of CP-1 and CP-2.

The Solid-State UV-vis Spectra of CP-1 and CP-2.

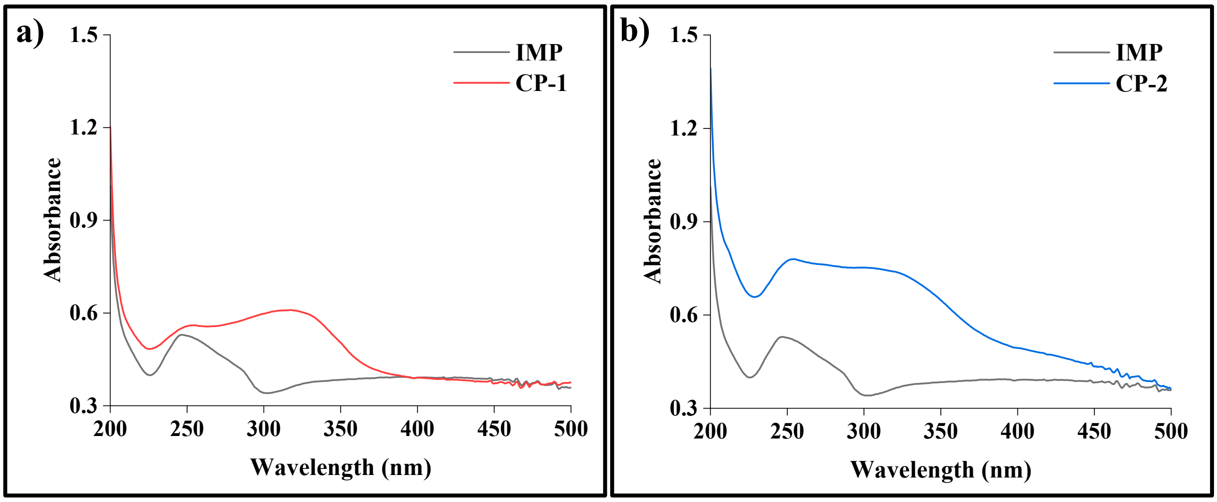

Figure. S19. UV–vis spectra of (a) IMP, bpe and CP-1 (d) IMP, azpy and CP-2. The spectra were obtained by measuring 5.0×10^{-5} mol·L^{-1} solution in a 1 cm cell.

The Thermo-Gravimetric Analysis of CP-1 and CP-2.

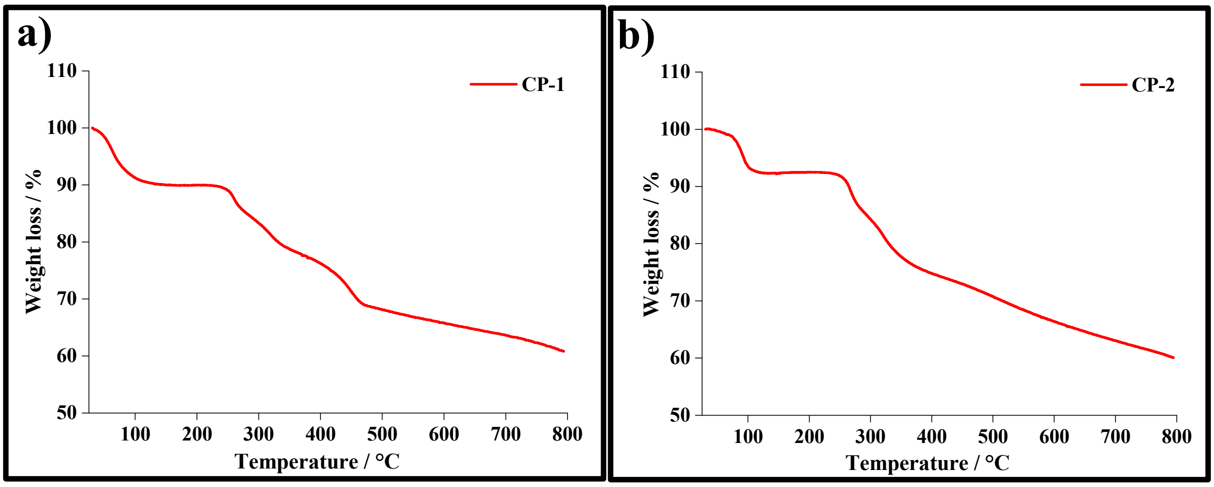

Figure S20. TGA curves of CP-1 and CP-2.

4. In Silico study of CP-1 and CP-2 with HU Drug

Table S8. The Molecular Docking interaction values of the CP-1 with the Drug (HU).

**Drug Atom (Hydroxy carbamide)**	**Coordination Polymer Atom (CP-1)**	**Distance (Å)**
H	O	2.63
N	H	2.98
H	O	2.63
N	H	2.97
N	H	2.94
H	N	2.81
H	O	1.53
N	H	3.11
O	H	2.52
H	O	2.55

Table S9. The Molecular Docking interaction values of the CP-2 with the Drug (HU).

Drug Atom (Hydroxycarbamide)	Coordination Polymer Atom (CP-2)	Distance (Å)
H	N	3.27
H	O	2.63
O	H	2.39
O	H	2.83
N	H	3.35
H	O	2.30
O	H	1.95
H	N	3.55
H	O	2.68
O	H	2.86

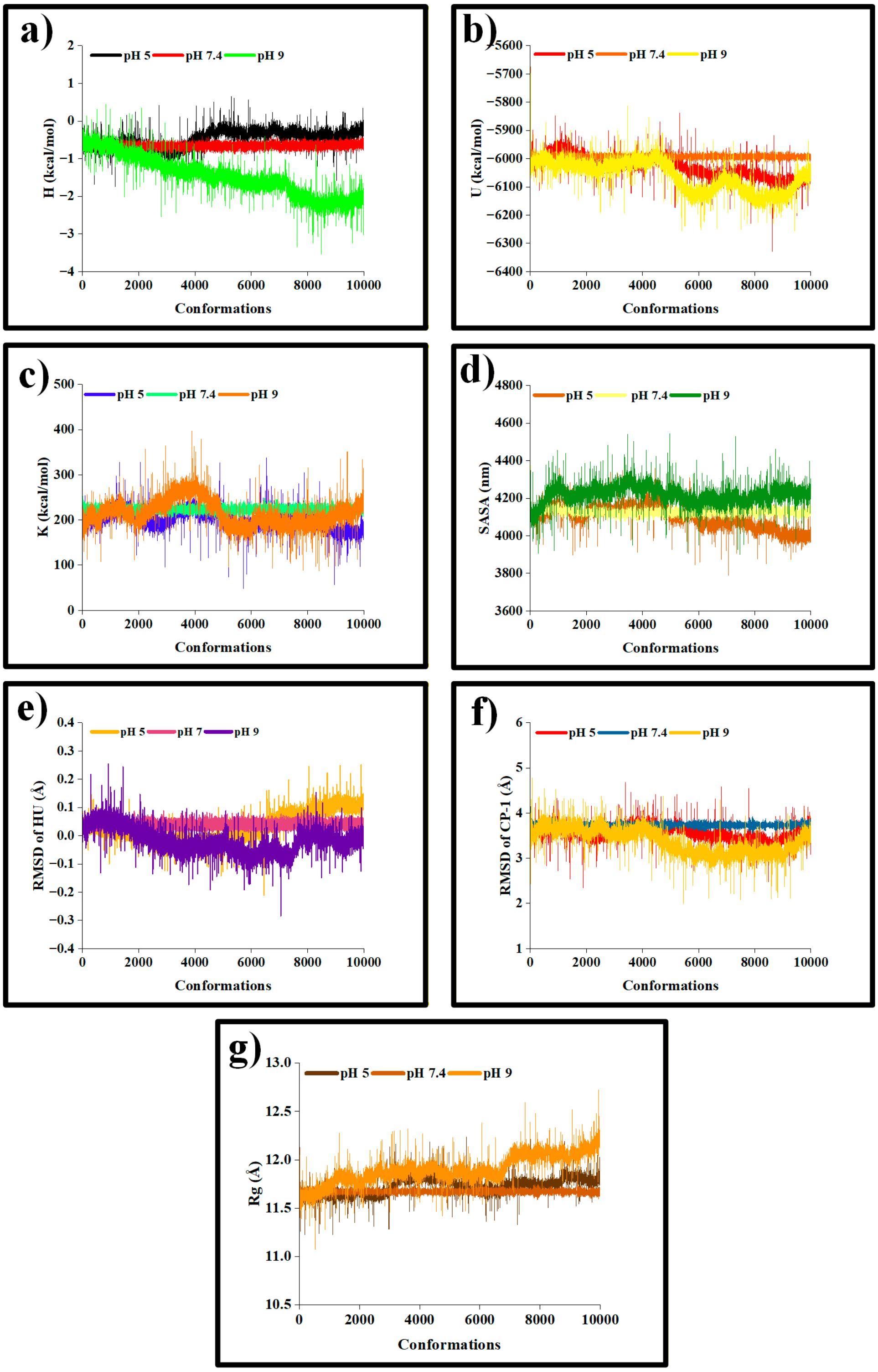

Figure S21. (a) This MD graph shows the enthalpy interactions of the Drug HU with the CP-1 with three different pH at 10,000 conformations. The spectra in red at the

blood pH 7.4 indicate more stable around -0.8 $kcalmol^{-1}$ with no drift. The black spectra shows more fluctuation at pH 5 which is least favourable. Green spectra represent the enthalpy gain −0.6 to −1.7 kcal mol^{-1}. **(b)** The potential energy graph between the drug and CP-1 is tell us that the orange spectra at pH 7.4 shows less noise and consistency of binding. The yellow spectra is at pH 9 indicated the downward shifting and fluctuation while red spectra at pH 5 is also shift downward with no stability. **(c)** This is the graph of Kinetic energy between the drug and CP-1 at different pH over 10,000 conformations. Green spectra at pH 7.4 shows minimal drift, most stable and tight packing between the drug and Cp-1. While the pH 5 (blue) and pH 9 (orange) shows less stability and repacking. **(d)** This is the graph of Solvent accessible surface area (SASA) between the drug and CP-1 at three pH over 1000 conformations. At pH 7.4 (light gold) shows stable graph around 4.1–4.2 × 10^3 which represent the consistency, most stationary and drug in a well define pocket of CP-1. While at pH 5 (brown) shows less stability but pH 9 (green) shows quite stability and increase solvent exposure. **(e)** RMSD graph of drug HU shows the binding of drug HU at different pH values across 10,000 conformations. The pink spectra denoted the pH 7 and according to this spectra the binding of drug and CP-1 is more rigid due to the lowest and smooth RMSD band with no drift**.** At the acidic pH 5 spectra is in gold and it shows the more fluctuations and broad band which is shifted to the upward at 6–7×10^3 confirmations. Moreover at the basic pH 9 which is in purple colour indicated the minute shifting between 5–9×10^3 conformations and it shows it is more stable as compared to pH 5. **(f)** RMSD graph of CP-1 is taken at different pH over 10,000 conformations. At pH 7.4 (blue) spectra exhibit low noise (3.7–3.9 Å), flat and stationary geometry. At the acidic pH 5 (red) indicated the intermediate fluctuation and mild compaction while at basic pH 9 (yellow) shows more fluctuation which indicate repacking of the framework. **(g)** Radius of gyration graph between drug HU and CP-1 indicated that at pH 7.4 (brown trace) less noise plateau around (11.6–11.8 Å) which shows the stationary pore geometry and dormancy. At pH 5 (dark brown) shows a mild compactness while at pH 9 (orange trace) shows more fluctuation and repacking at (12.1–12.3 Å).

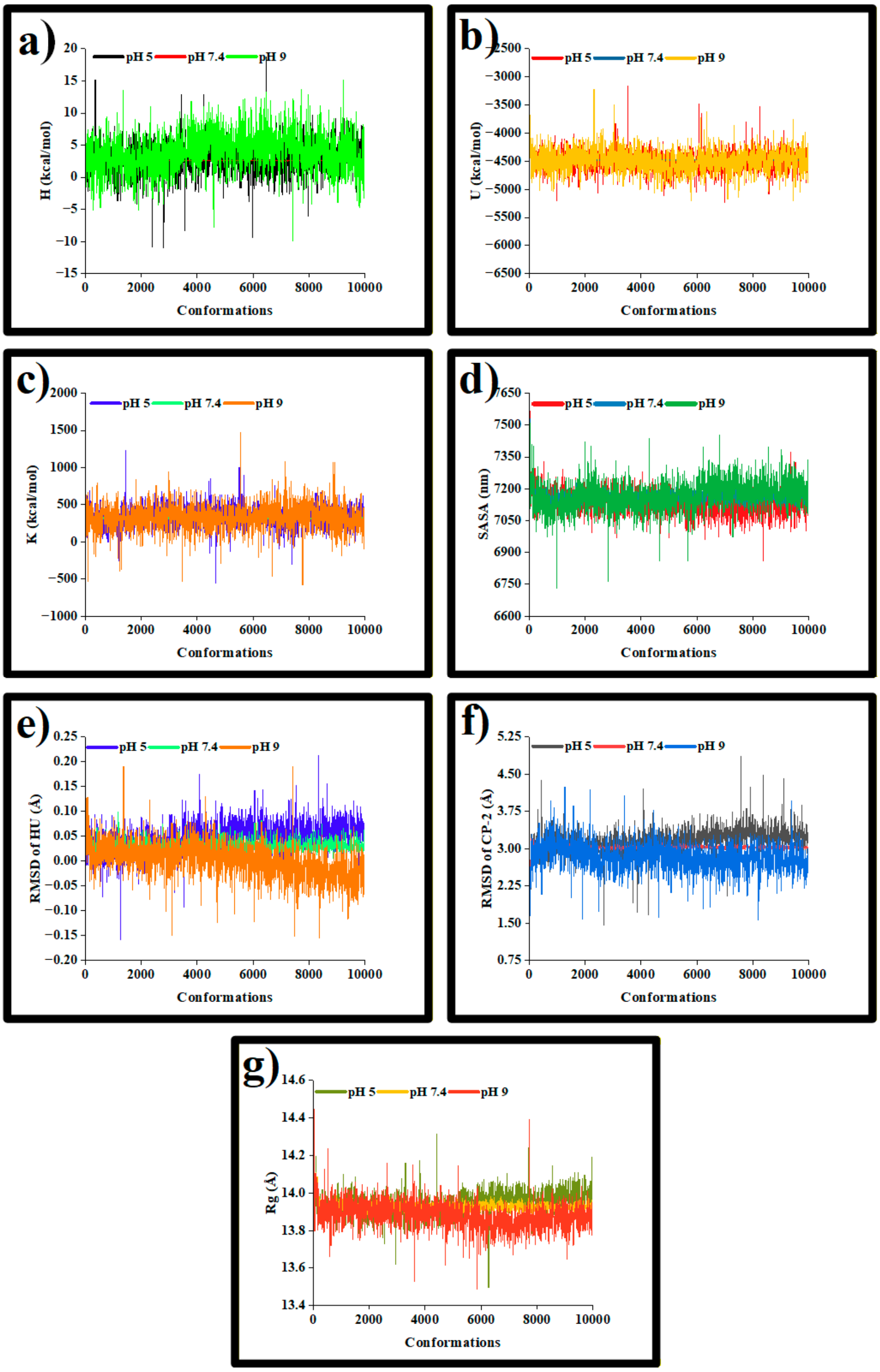

Figure S22. **(a)** Graph shows the enthalpy interactions between the CP-2 and drug HU. At pH 7.4 (red) spectra remains almost stationary while at pH 5 (black) spectra

shows the minimum distribution and pH 9 (green) indicate the highest fluctuation and repacking. **(b)** Potential energy graph between CP-2 and Drug at different conformations with different pH. At pH 7.4 (blue) spectra exhibit the slightly more negative and consistent bonding. Red spectra at pH (5) shows some fluctuations and not consistency while at pH 9 (yellow) indicate the less negative and more spreading with minimum pore. **(c)** Graph shows the kinetic energy versus the conformations. The neutral spectra at pH 7.4 (green) indicated that the minute distribution while acidic spectra at pH 5 (blue) shows a comparable mean and at pH 9 (orange) spectra gives the highest variance and repacking. **(d)** Graph tells about the solvent accessible surface area (SASA) of CP-2 and Drug HU over 10000 conformations with different pH. At pH 7.4 (blue) spectra is steady with a stable pocket. Red spectra at pH 5 is lower means more compact pocket with drug. The basic pH 9 (green) shows spectra is the highest and fluctuate exposed the drug with more pockets. **(e)** RMSD graph of Drug against the conformation at different pH. Green spectra (pH 7.4) shows the less stability as compared to pH 9 (orange) due to the higher band. While pH 5 (blue) spectra indicate a lot of dispersion and show least stability. **(f)** RMSD graph of CP-2 against 10000 conformations at three different pH. pH 7.4 the spectra in red shows the weak band as compared to other pH. Spectra in grey at pH 5 indicate the spikes to upward expansion while at pH 9 (blue) exhibit fast motion near the guest. **(g)** Rg graph shows that yellow spectra is moderate at pH 7.4 due to the weak band while spectra (green) at pH 5 has higher Rg value which denoted the very big cavities. Moreover pH 9 (red) spectra is the most stable with no drift and narrow band at 13.8-14 Å present the host guest stable structure.

5. HIRSHFIELD SURFACE ANALYSIS PLOTS

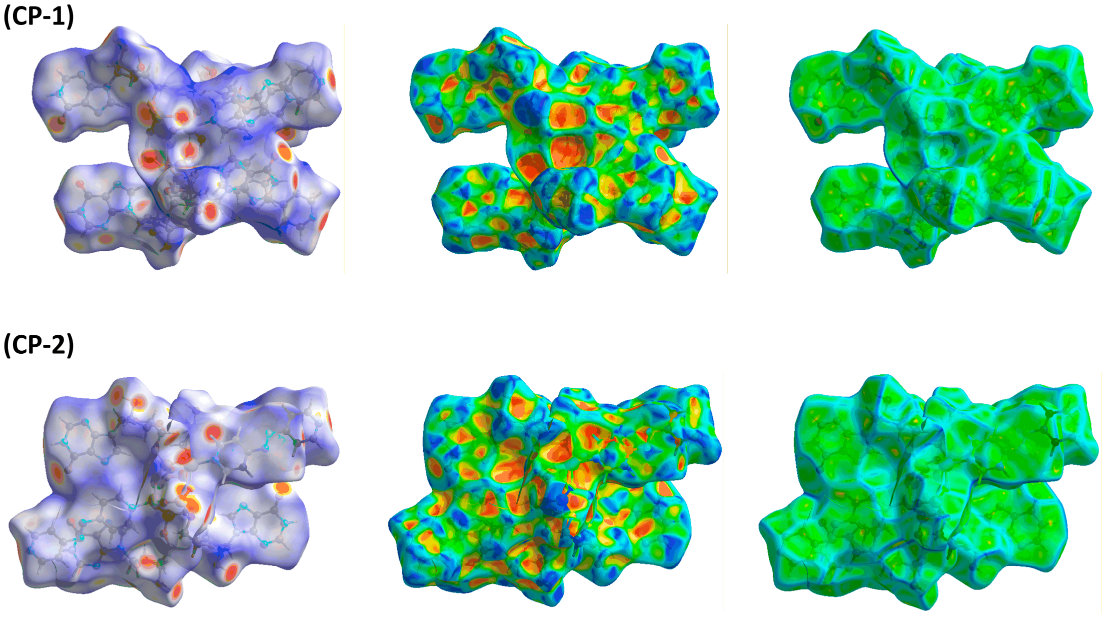

Figure S23. Views of the three-dimensional Hirshfeld surfaces of **CP-1** and **CP-2**, with dnorm plots, shape indexes, and curvedness.

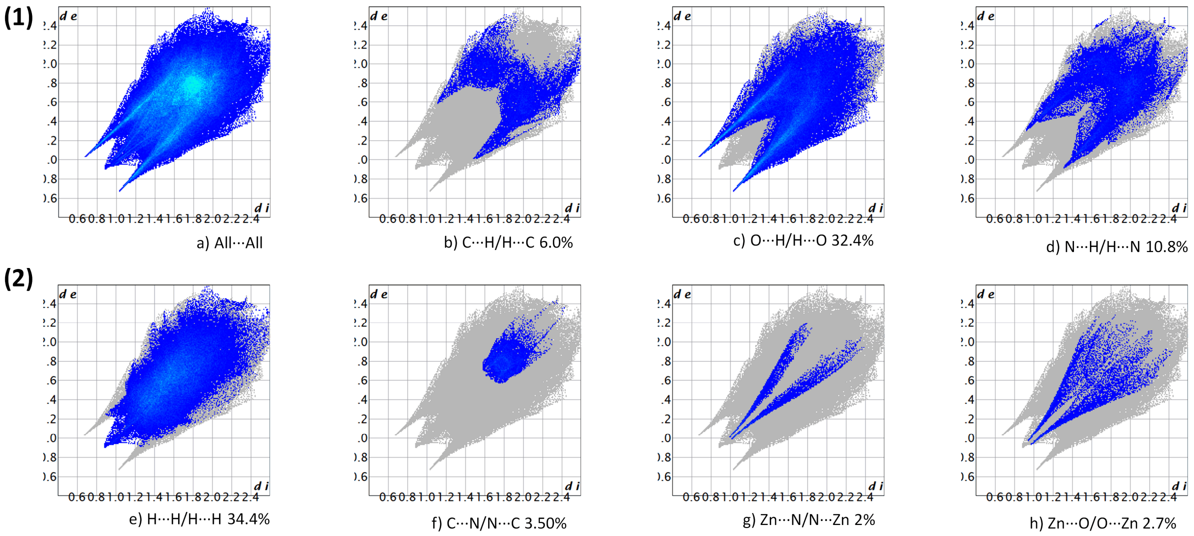

Figure S24. The full two-dimensional fingerprint plots for CP-1. The di and de values are the closest internal and external distances (in Å) from given points on the Hirshfeld surface.

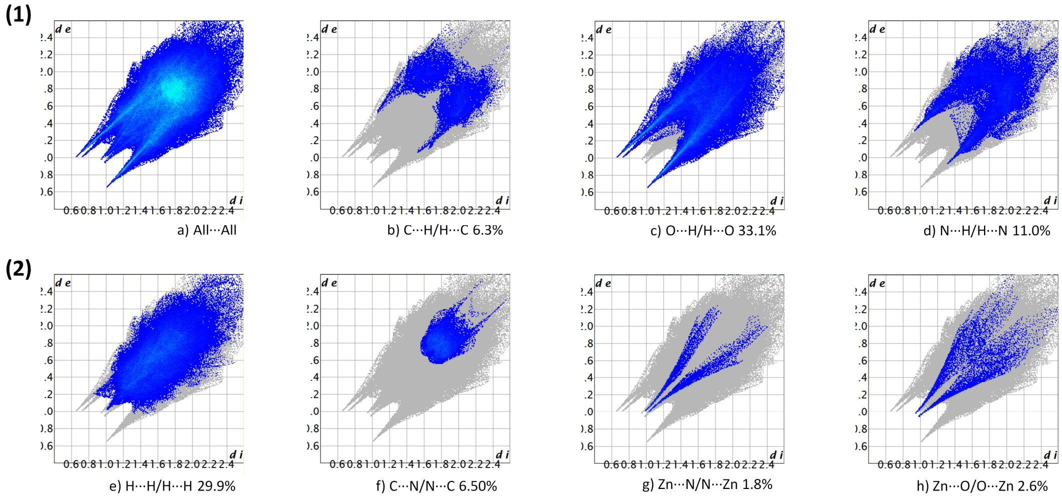

Figure S25. The full two-dimensional fingerprint plots for CP-1. The di and de values are the closest internal and external distances (in Å) from given points on the Hirshfeld surface.

Refrences

1. Zhu, Y.; Li, Z.; Song, W.; Khan, M. A.; Li, H., Conformation locking of the pentose ring in nucleotide monophosphate coordination polymers via π–π stacking and metal-ion coordination. *Inorganic Chemistry* **2021,** *61* (2), 818-829.
